# Umbrella Review of Systematic Reviews and Meta-analyses on Consumption of Different Food Groups and Risk of All-cause Mortality

**DOI:** 10.1016/j.advnut.2025.100393

**Published:** 2025-02-15

**Authors:** Anindita Tasnim Onni, Rajiv Balakrishna, Matteo Perillo, Marco Amato, Elaheh Javadi Arjmand, Lise M Thomassen, Antonello Lorenzini, Lars T Fadnes

**Affiliations:** 1 Department of Global Public Health and Primary Care, University of Bergen, Bergen, Norway; 2 Department of Addiction Medicine, Haukeland University Hospital, Bergen, Norway; 3 Department of Biomedical and Neuromotor Sciences, University of Bologna, Bologna, Italy; 4 Biostructures and Biosystems National Institute (INBB), Rome, Italy

**Keywords:** food, nutrition, diet, food groups, mortality, life expectancy, meta-analysis, systematic review

## Abstract

Consumption of different food groups is linked to a range of health outcomes. It is essential to integrate the most reliable evidence regarding intake of different food groups and risk of mortality to optimize dietary guidance. Our aim is to systematically and comprehensively assess the associations between the consumption of various food groups and all-cause mortality. The food groups under consideration include edible grains (refined and whole grains), fruits, vegetables, nuts, legumes, fish and fish products, eggs, dairy products/milk, meat and meat products (including processed meat, unprocessed red and white meat), sugar-sweetened beverages, and added sugars. We present these associations with high compared with low consumption and per serving comparisons. We comprehensively and systematically reviewed a search in Medline, Embase, Web of Science, and Epistemonikos (PROSPERO: CRD42024498035), identifying 41 meta-analyses involving over a million participants, many of which showed significant heterogeneity. Of the 41 studies, 18 were rated high quality, 8 moderate quality, 5 low quality, and 10 critically low quality according to AMSTAR-2 assessments. Our findings revealed that higher consumption of nuts, whole grains, fruits, vegetables, and fish was associated with lower mortality rates, both in high compared with low comparisons and per serving analyses. Similarly, we observed favorable outcomes for legumes and white meat in high compared with low comparisons. Conversely, high intakes of red and processed meats, as well as sugar-sweetened beverages, were linked to higher all-cause mortality. Dairy products and refined grains did not show clear associations with mortality, whereas there was a tendency in all-cause mortality for high intakes of added sugars and eggs.

**Statement of significance**

This umbrella review provides current, comprehensive, and up-to-date evidence regarding the association between various food groups and all-cause mortality. Additionally, it examines dose-response relationships and provides insights into intermediate factors affecting chronic disease outcomes.

## Introduction

Non-communicable diseases are responsible for around two-thirds of all deaths globally, with ischemic heart disease as the leading cause of death [1]. Dietary patterns play an essential role in the development of non-communicable diseases and cause-specific mortalities [[2], [3], [4], [5], [6]]. Each food group has a distinct nutritional profile that may affect longevity, partly mediated by metabolic and cardiovascular disease risk factors such as high systolic blood pressure, high BMI, and high fasting plasma glucose [7]. Because suboptimal dietary patterns are preventable risk factors for chronic non-communicable diseases and their mortality, understanding how different food groups are associated with mortality is crucial when revising dietary guidelines and informing public health measures, such as promotion and setting of taxation on different foods [2,6]. For instance, nuts, known for their healthy fats, proteins, and bioactive compounds, have been associated with a reduced mortality and several morbidities [8]. Similarly, fruits and vegetables have a high content of essential vitamins, phytochemicals, and dietary fibers, which are likely to contribute to their associations to lower rates of all-cause mortality and key morbidities [9,10]. Conversely, excessive consumption of sugar-sweetened beverages and processed meats are associated with an increased mortality risk and diabetes type 2 [11,12].

All-cause mortality is a measure that is generally more robust and less prone to misclassification than disease-specific mortalities, because many deaths are not solely attributable to a single disease, and the main cause of death is not always obvious [13,14]. Furthermore, all-cause mortality can be considered the total of various cause-specific mortalities. The association between eating and all-cause mortality thus provides an overall estimate of potential longevity-related gains or losses because of dietary changes [4]. With a quick rise in the number of meta-analyses during the last decade, there are around 50 meta-analyses on individual food groups and their associations with mortality. However, there is currently no up-to-date systematic umbrella review that integrates these. Thus, an overall overview of food groups and their association with mortality could help guide dietary guidelines and public health measures.

The aim of this umbrella review is to systematically analyze evidence from systematic reviews and meta-analyses on the impact of different food groups on risk of all-cause mortality. We investigated the associations between food groups, such as edible grains (refined and whole grains), fruits, vegetables, nuts, legumes, fish and fish products, eggs, milk/dairy products, meat and meat products (including processed meat, unprocessed red and white meat), sugar-sweetened beverages, and added sugars, and risk of mortality by examining data from prospective studies. We assess both high compared with low comparisons and per-serving and non-linear dose-response relationships.

## Methods

To summarize the evidence from meta-analyses and systematic reviews regarding the consumption of various food groups, we assessed their association with all-cause mortality using an umbrella review framework [15]. The study protocol is registered with PROSPERO (https://www.crd.york.ac.uk/prospero/display_record.php?ID=CRD42024498035). This systematic review was conducted in accordance with the PRISMA guidelines [16]. For additional details regarding the search methodology, see Supplemental Text 1.

### Eligibility criteria

#### Types of studies

We evaluated meta-analyses based on systematic reviews that included analyses from cohort studies, case-control studies, and randomized controlled trials regarding the consumption of various food groups and their associations with all-cause mortality. Detailed inclusion and exclusion criteria are outlined in Box 1. Studies with cross-sectional designs or those providing only regional estimates not generalizable to broader populations were excluded. In the case that the systemic reviews included both cross-sectional and cohort designs, we extracted the values reported only for cohort studies. No restrictions were applied to the publication date, but only articles written in English were considered.

BOX 1 **Inclusion and exclusion criteria**

**Table:** 

Inclusion criteria:
• Study types: meta-analysis based on systematic reviews presenting analyses from longitudinal observation studies (e.g., cohorts, case-control), and trials
• Exposure: consumption of any of these 14 food groups: edible grains (refined and whole grains), fruits, vegetables, nuts, legumes, fish and fish products, eggs, dairy products and milk, meat and meat products (including processed meat, red unprocessed meat, and white unprocessed meat), sugar-sweetened beverages, and added sugars
• Comparators: high vs. low consumption, and dose-response relationship between exposure and outcomes
• Outcome: all-cause mortality and life expectancy
• Language: English
• Publication status: published articles indexed in Medline, Embase, Web of Science, and Epistemonikos from inception to 19 November, 2024
Exclusion criteria:
• Study types: meta-analyses based on non-systematic reviews (e.g., reviews in which search was conducted in only 1 repository), systematic reviews without meta-analyses, meta-analyses including cross-sectional studies
• Population: meta-analyses providing only regional estimates, covering only specific sub-populations (e.g., only people affected by a specific disease or type of diseases), or including children and adolescents; animal studies
• Exposure: studies only reporting on macro- or micronutrients

Alt-text: BOX 1

### Search

In collaboration with an experienced librarian, a total of 4220 records were retrieved from the databases Medline, Embase, Web of Science, and Epistemonikos. The search covered the period from database inception to 19 November, 2024. After automatic deduplication using EndNote 21, 2240 records remained. No restrictions were applied to the publication date.

The search included the terms listed below. Articles were retrieved if they covered ≥1 of the elements for each of the points.

1) Edible grain (refined and whole grains), fruit, vegetables, nuts, legumes, pulses, fish, fish products, eggs, milk and dairy, meat and meat products (including processed and unprocessed red and white meat), sugar-sweetened beverages, or added sugars.
2) Intake, consumption, eat, or diet.
3) Systematic review and meta-analysis.
4) Mortality, cause of death, fatal outcome, longevity or life expectancy.

### Data collection process and data extraction

All imported references were screened in duplicate by 3 authors using the tool Rayyan. Screening involved reviewing titles and abstracts, followed by a full-text assessment of articles deemed potentially relevant. All relevant articles were independently read in full text in duplicate by ≥2 of the authors (ATO, RB, MA) involved in the process, and all extractions were double-checked. Any discrepancies in screening and extraction were discussed and resolved by consensus. Furthermore, another researcher (MP) performed a set of simulations of the titles and abstracts’ screening using the software ASReview, which exploits active learning and natural language processing to streamline the process of titles and abstracts screening [17]. The screening simulations were performed using ASReview v1.5 [18], and allowed us to estimate how much time would have been saved and how much the recall of relevant articles would have changed in case the screening had been performed by the same reviewers using ASReview with different settings. More information about the simulations is available in Supplemental Text 2.

Data considered relevant were extracted into a Microsoft Excel table. Information gathered included: first author, search year, number of primary studies, number of participants, number of observed deaths, estimated association with all-cause mortality (only for high compared with low and linear dose-response: point estimate risk ratio or hazard ratio (HR), as well as 95% confidence interval), heterogeneity for high compared with low and linear dose-response, intake range for the non-linear dose-response analysis, AMSTAR-2 score. All extracted data values were double-checked by the authors.

### Risk of bias in individual studies and across studies

The risk of bias was assessed using the AMSTAR-2 tool [19]. Reviews were categorized as high, moderate, low, or critically low quality based on this tool. Each study was independently rated by 1 author from a team of 3 authors (ATO, MA, RB), and their assessment was subsequently checked by another author from a different team of 3 authors (LTF, MP, AL). Any discrepancies were discussed and resolved by consensus. Details of these assessments are provided in Supplemental Table 1.

### Selection of the most relevant meta-analyses

For systematic reviews, cohorts included in the different meta-analyses could overlap when they share exposures and outcomes. The most comprehensive and recent meta-analysis had generally included all the studies reporting in the previous and less comprehensive ones. For each combination of food groups and type of comparison (high compared with low and per serving), the most recent and comprehensive meta-analysis was identified. This was done by selecting the most recent among the meta-analyses that had the highest number of studies, participants, or observed deaths. The studies graded as low quality were excluded by the pool of candidates' most recent and comprehensive meta-analyses. However, we included some moderate- or low-quality evidence in cases where no high-quality meta-analyses were available for a specific combination of food group and comparison. The details of the decision tree process are presented in Supplemental Tables 2 and 3.

### Analysis

Tables were created to present the data extracted from the included studies, as well as the AMSTAR-2 assessment of such studies. These data were summarized in figures visualizing the associations between food groups and all-cause mortality both for high compared with low comparison and dose-response. A table was generated for each combination of food group and comparison (high compared with low, linear dose-response, and non-linear dose-response). For per serving, we also report a converted HR to make it comparable across studies [HR_standardized_ = HR_reported_^(standardized dose/reported dose)^, and similarly for the confidence intervals]. The standard serving sizes are reported in Table 1 [2,9,11,12,[20], [21], [22], [23], [24], [25]]. The standardized dose-response was defined using the following serving sizes: added sugars: 10 g/d, dairy products: 200 g/d (in milk equivalents), eggs: 50 g/d, fish: 100 g/d, fruits: 80 g/d, legumes: 50 g/d, nuts: 28 g/d, processed meat: 50 g/d, unprocessed red meat: 100 g/d, refined grains: 30 g/d (product weight/fresh weight), sugar-sweetened beverages: 250 g/d or 250 mL/d, vegetables 100 g/d, white meat: 100 g/d, and whole grains: 30 g/d (product weight/fresh weight). For studies that reported intake solely as serving size without specifying quantitative amounts, standard recommended conversions were applied. For high compared with low and per serving comparison, the same data were also used to produce forest plots visually summarizing the associations reported in the meta-analysis. We present a table and 2 forest plots reporting characteristics and the results of the most recent and comprehensive meta-analyses (1 for each combination of exposure and comparison). Plots presenting all meta-analyses including those not considered most recent and most comprehensive are available in the Supplemental Materials. The data preprocessing, the conversion of the results, and the generation of tables and plots were conducted using Studio R 4.4.1.

**TABLE 1:** Overview of the most comprehensive/up-to-date meta-analyses on associations between food groups and all-cause mortality with details on first author, search year, reference number, exposure, number of studies, participants, mortalities, quality (AMSTAR-2) summary estimates, and heterogeneity.

Author, year (ref.no.)	Food group	Comparison	Studies/participants/deaths	AMSTAR-2	Hazard ratios [CI], (*I*^2^)
Huang, 2022 [20]	Added sugars	HL	9/669,068/62,868	Moderate	1.05 [0.97, 1.14] (*I*^2^ = 0.77)
Huang, 2022 [20]	Added sugars	PS (10%E)	10/680,444/63,709	Moderate	1.03 [0.97, 1.08] (*I*^2^ = 0.72)
Schwingshackl, 2016 [2]	Dairy	HL	27/938,817/126,759	High	1.03 [0.98, 1.07] (*I*^2^ = 0.94)
Schwingshackl, 2016 [2]	Dairy	PS (200g)	16/546,894/93,597	High	0.98 [0.93, 1.03] (*I*^2^ = 0.96)
Ma, 2021 [21]	Eggs	HL	25/1,477,561/240,552	Moderate	1.04 [0.99, 1.09] (*I*^2^ = 0.81)
Yang, 2021 [22]	Eggs	PS (50g)	19/1,737,893/214,073	High	1.08 [1.01, 1.15] (*I*^2^ = 0.91)
Schwingshackl, 2016 [2]	Fish	HL	39/1,671,191/157,688	High	0.95 [0.92, 0.98] (*I*^2^ = 0.51)
Schwingshackl, 2016 [2]	Fish	PS (100g)	19/1,072,352/94,132	High	0.93 [0.88, 0.98] (*I*^2^ = 0.53)
Kazemi, 2020 [23]	Fruit	HL	28/1,626,395/108,402	Moderate	0.89 [0.85, 0.93] (*I*^2^ = 0.90)
Kazemi, 2020 [23]	Fruit	PS (80g)	22/1,582,256/89,773	Moderate	0.96 [0.94, 0.98] (*I*^2^ = 0.87)
Schwingshackl, 2016 [2]	Legumes	HL	17/603,420/53,085	High	0.96 [0.93, 1.00] (*I*^2^ = 0.48)
Schwingshackl, 2016 [2]	Legumes	PS (50g)	6/386,116/24,882	High	0.96 [0.90, 1.01] (*I*^2^ = 0.48)
Schwingshackl, 2016 [2]	Nuts	HL	16/866,312/80,204	High	0.80 [0.74, 0.86] (*I*^2^ = 0.84)
Schwingshackl, 2016 [2]	Nuts	PS (28g)	16/902,178/80,204	High	0.76 [0.69, 0.84] (*I*^2^ = 0.82)
Zeraatkar, 2018 [12]	Processed meat	HL	10/696,822/NA	High	1.14 [1.10, 1.18] (*I*^2^ = 0.17)
Zeraatkar, 2018 [12]	Processed meat	PS (50g)	8/1,241,900/NA	High	1.22 [1.10, 1.38] (*I*^2^ = 0.87)
Schwingshackl, 2016 [2]	Red meat	HL	12/1,762,627/177,655	High	1.10 [1.00, 1.20] (*I*^2^ = 0.93)
Schwingshackl, 2016 [2]	Red meat	PS (100g)	10/1,388,587/151,912	High	1.10 [1.04, 1.18] (*I*^2^ = 0.92)
Hu, 2022 [24]	Refined grains	HL	3/174,512/13,943	High	1.12 [0.95, 1.31] (*I*^2^ = 0.71)
Hu, 2022 [24]	Refined grains	PS (30g)	5/176,957/15,444	High	1.00 [0.97, 1,02] (*I*^2^ = 0.88)
Li, 2022 [11]	SSBs	HL	14/1,131,605/121,488	High	1.11 [1.05, 1.16] (*I*^2^ = 0.75)
Li, 2022 [11]	SSBs	PS (250g)	14/1,131,605/121,488	High	1.08 [1.06, 1.09] (*I*^2^ = NA)
Schwingshackl, 2016 [2]	Vegetables	HL	37/1,409,290/121,067	High	0.93 [0.90, 0.95] (*I*^2^ = 0.75)
Aune, 2016 [9]	Vegetables	PS (100g)	22/1,050,795/86,264	High	0.93 [0.91, 0.96] (*I*^2^ = 0.82)
Papp, 2021 [25]	White meat	HL	12/1,435,390/120,122	High	0.96 [0.93, 0.98] (*I*^2^ = 0.05)
Papp, 2021 [25]	White meat	PS (100g)	11/1,138,669/95,839	High	0.98 [0.94, 1.02] (*I*^2^ = 0.19)
Schwingshackl, 2016 [2]	Whole grains	HL	19/1,044,464/121,141	High	0.88 [0.84, 0.92] (*I*^2^ = 0.91)
Hu, 2022 [24]	Whole grains	PS (30g)	13/912,293/106,112	High	0.94 [0.92, 0.97] (*I*^2^ = 0.90)

Abbreviations: CI, confidence interval; HL, high compared with low; PS, per serving; SSB, sugar-sweetened beverage.

## Results

Of the 2240 screened articles, 53 were considered potentially relevant after the titles and abstracts screening. The simulations with ASReview showed that, using such a tool, all the screeners would have saved ≤75% of their screening time without missing any relevant records, while also reducing the number of conflicts to discuss. Supplemental Text 2 provides more details about the performance observed in the simulations and the optimal settings for ASReview in this specific context.

**FIGURE 1:**
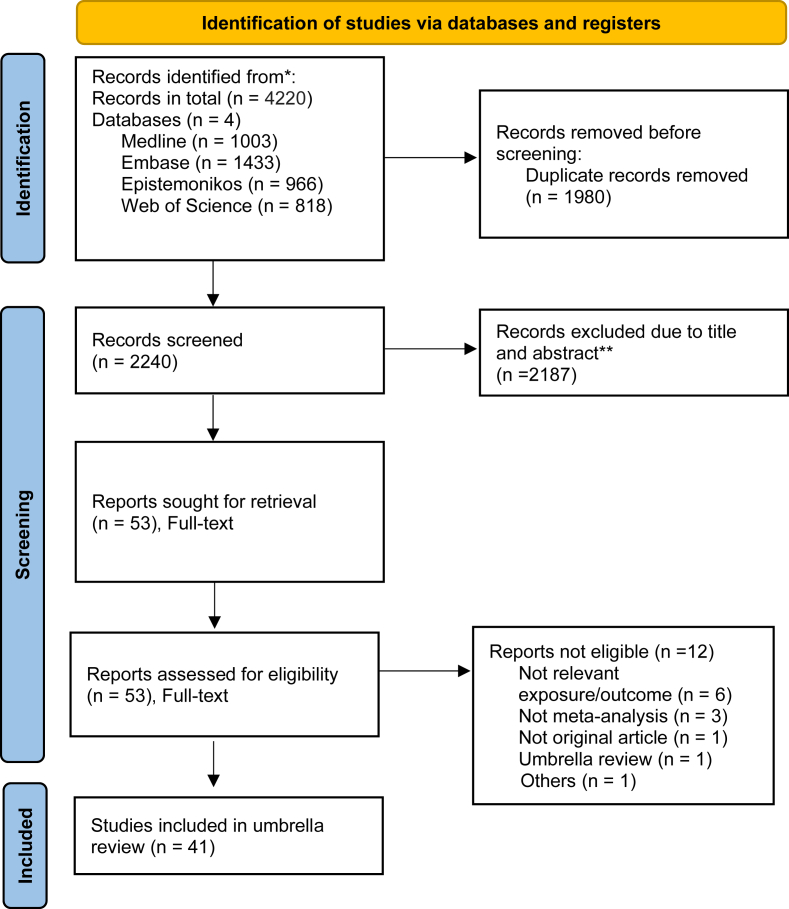
PRISMA flowchart. ∗The number of records identified from each database or register searched are Medline (*n* = 1003), Embase (*n* = 1433), Epistemonikos (*n* = 966), and Web of Science (*n* = 818). ∗∗No automation tool was used, indicating 2187 records were excluded by a human. Source: Page MJ, et al. BMJ 2021;372:n71. https://doi.org/10.1136/bmj.n71. This work is licensed under CC BY 4.0. To view a copy of this license, visit https://creativecommons.org/licenses/by/4.0/.

After the full-text screening, 12 studies were excluded because not having relevant exposure and outcome, were not meta-analyses or original articles, or were umbrella reviews [[26], [27], [28], [29], [30], [31], [32], [33], [34], [35], [36], [37]]. In total, 41 articles were included for the present review, containing a total of 115 exposure and outcome combinations from meta-analyses on the associations between individual food groups and all-cause mortality (Figure 1) [2,9,11,12,[20], [21], [22], [23], [24], [25],[38], [39], [40], [41], [42], [43], [44], [45], [46], [47], [48], [49], [50], [51], [52], [53], [54], [55], [56], [57], [58], [59], [60], [61], [62], [63], [64], [65], [66], [67]]. The most recent and comprehensive meta-analyses were conducted between 2016 and 2022, depending on the food group (Table 1). For most food groups, the combined data included over a million participants, with some analyses encompassing >200,000 deaths, providing a solid basis for robust conclusions [21,22]. However, certain food groups, such as refined grains, had fewer participants (174,512) and recorded events (13,943 deaths), suggesting that the evidence for these groups may be less robust [24]. Most of the studies showed significant heterogeneity, with >80% displaying a heterogeneity *I*^2^ value >0.5, indicating the diverse nature of the associations between dietary factors and mortality outcomes. Less heterogeneity was seen for legumes (in both high compared with low and dose-response comparisons), processed meat (high compared with low), and white meat (both high compared with low and dose-response) [2,12,25]. All the articles included cohorts, whereas none had intervention or case-control designs. For high compared with low comparisons, the median number of cohort studies included per meta-analysis was 16.5 (ranging from 3 to 39). For per-serving comparisons, the median number of cohort studies included per meta-analysis was 13.5 (ranging from 5 to 22).

**FIGURE 2:**
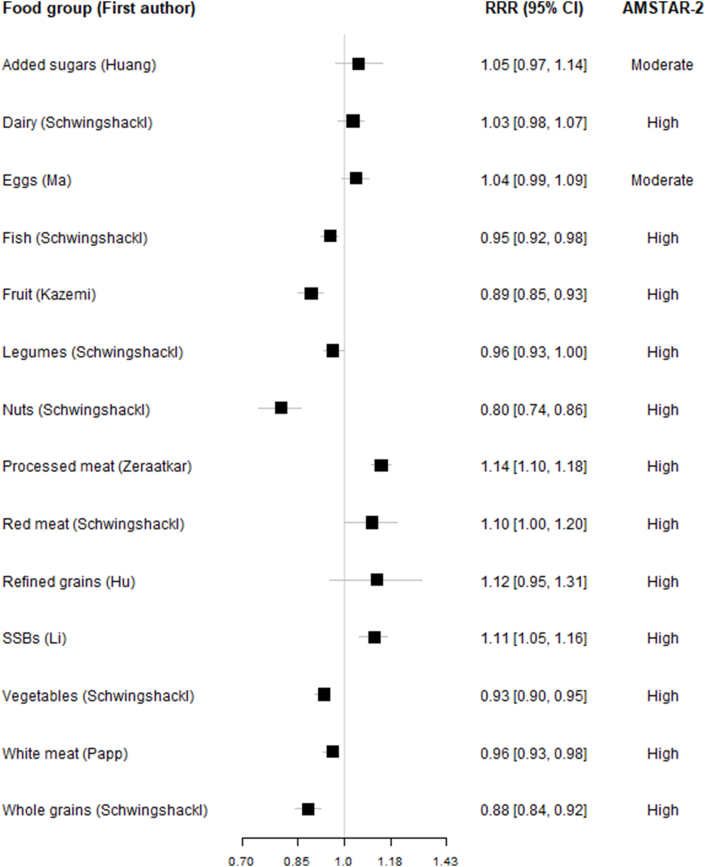
Associations between food groups (high vs. low consumption) and all-cause mortality in most comprehensive and up-to-date meta-analyses.

**FIGURE 3:**
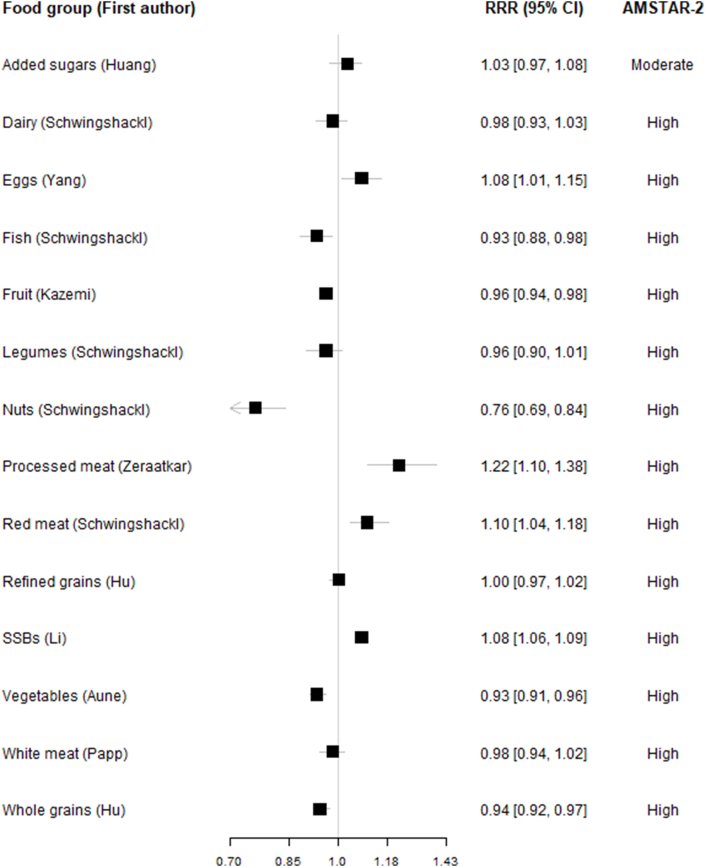
Associations between food groups (per serving) and all-cause mortality in most comprehensive and up-to-date meta-analyses.

The meta-analyses presenting high compared with low intake comparisons (Figure 2) and dose-response comparisons (Figure 3) indicated associations between high intakes of nuts, whole grains, fruits, vegetables, and fish, and reduced all-cause mortality. Conversely, a higher intake of processed meat, unprocessed red meat, and sugar-sweetened beverages were associated with increased mortality risks. For legumes and white meat, a higher intake tended to be associated with lower mortality. Conversely, non-significant tendencies to higher mortality were observed for added sugars, eggs, and refined grains. The evidence regarding the diary showed no clear associations with mortality. We also identified studies on non-linear dose-response associations of the food groups and all-cause mortality, and it showed similar characteristics as per serving. Additional descriptions are provided in Supplemental Text 3.

Among the 41 included studies, 18 were assessed as high quality using the AMSTAR-2 tool, 8 as moderate quality, 5 as low quality, and 10 as critically low quality [19]. The quality criteria most typically lacking were lack of registered protocol (12 studies), not reporting funding of included studies (31 studies), not specifying duplicate screening (10 studies), not using tools for confounding (6 studies), and not presenting sensitivity analysis (6 studies) (Supplemental Table 1). The most recent and comprehensive meta-analyses were predominantly rated as high quality, with a few exceptions classified as moderate quality (added sugar for high compared with low and per serving, egg for high compared with low, and fruits for high compared with low and per serving). Additional meta-analyses, along with their detailed findings, can be found in Supplemental Tables 2–6 and Supplemental Figures 1–28.

## Discussion

This umbrella review provides a comprehensive analysis of the relationships between various food groups and all-cause mortality. Our results support the associated longevity benefits from increased consumption of nuts, whole grains, fruits, vegetables, and fish—all clearly associated with lower mortality rates with increasing intakes. These foods are generally abundant in crucial nutrients, including vitamins, minerals, dietary fiber, and healthy fats, all of which are essential for minimizing inflammation, enhancing metabolic health, and preventing non-communicable diseases such as cardiovascular disease, type 2 diabetes, and cancers [6,8,[68], [69], [70], [71], [72], [73], [74], [75]], which are the likely mediators of the inverse associations with mortality.

Foods such as whole grains, fruits, vegetables, and nuts are abundant in fiber, antioxidants, and bioactive substances that help reduce oxidative stress and inflammation [8,39,76], contributing to boosting immune function and endothelial functions [39,77]. This is also mirrored in decrease biomarkers such as C-reactive protein and IL-6, lipid levels, glucose and glycated hemoglobin, and enhanced gut health through the gut microbiome [5,[78], [79], [80], [81], [82], [83], [84], [85], [86]]. This is also strengthened by research on animals, indicating that foods high in polyphenols, such as berries and leafy greens, can inhibit tumor growth by boosting antioxidant defenses and lowering pro-inflammatory cytokines [87,88]. Similar outcomes are observed, where diets abundant in antioxidants contribute to a decrease in oxidative stress markers and enhancing protective antioxidants [89]. Studies on plant-dominated diets have generally demonstrated decreases in oxidative stress and inflammation, potentially lowering the mortality rates linked to these chronic diseases [90].

In contrast, a greater consumption of processed meats, unprocessed red meats, and sugar-sweetened beverages is associated with increased risk of mortality [[90], [91], [92], [93], [94], [95]]. Both high levels of saturated fats found in red and processed meats and high levels of free sugars are associated with inflammation, insulin resistance, and oxidative stress [[95], [96], [97]]. Chronic inflammation and oxidative stress are well-known processes that promote the onset of cardiovascular diseases, cancers, and several other disease groups [75,[98], [99], [100], [101], [102], [103]]. For some food groups such as dairy, the picture is more mixed with possibly inverse associations with low to moderate intakes and positive associations with higher intakes [2].

A challenge with the current evidence is the considerable heterogeneity observed among studies, especially in the meta-analyses that compare high compared with low intakes and dose-response evaluations. This variability might be influenced by differences in population characteristics, variation in the tools and methods for assessing dietary intake, and the range of foods within each food group category [2]. For instance, the food group vegetables consist of a variety of foods with differing nutrient compositions ranging from tomatoes, salads, potatoes, onion, and avocados, which have substantial differences in phytochemicals and even macronutrient profiles. This is likely to contribute to heterogeneity with different associations with mortality across studies depending on which of the vegetable “profile” within each group. For many food categories, there is insufficient research that allows for accurate estimates for subgroups within each category. Nonetheless, certain food groups, such as fruits and vegetables, tend to show stronger links to mortality when examining subgroups like tomatoes, leafy greens, and citrus fruits [9]. In contrast, the differences among subgroups appear to be less significant for food categories like nuts and whole grains, where the connections to mortality are more uniform across different types [8,39,104]. Future studies should aim to determine which specific foods within these groups provide the most health advantages, particularly in situations where significant variability remains. Future studies should aim to delve deeper into the distinct impacts of subcategories within each food group, especially in areas where variability remains significant. More detailed evaluations of the health effects associated with various preparation methods, portion sizes, and specific food items are necessary to enhance dietary guidelines [2,9]. Furthermore, although this review emphasizes several strong associations, there is a range in evidence quality. Some are from large and well-designed cohorts and supported by controlled trials with biomarkers of diseases, whereas others are on weaker evidence. Whole grains are on the high end of the meta-evidence with eggs on the other end, with most of the others being in between [2].

This study has strengths and limitations. It is the most comprehensive umbrella review to date on the relationships between food groups and all-cause mortality, with no other umbrella reviews of similar scope currently available. We followed a pre-registered PROSPERO protocol and adhered to PRISMA guidelines, ensuring methodological rigor. However, limitations in indexing within databases such as Medline, Embase, Web of Science, and Epistemonikos, or unclear indications of relevance in certain article titles and abstracts, may have resulted in some relevant data being missed. The selection of meta-analyses (over others) also introduces risk of a limitation because it is possible that another selection algorithm of meta-analyses could have provided slightly different results. However, results were generally similar across meta-analyses with overlapping exposures. Another important aspect of this study is that it allows us to validate the performance of ASReview as a supporting tool for articles screening in the specific setting of an umbrella review about the association between food group consumption and health outcomes. The very promising results obtained pave the way toward a more consistent use of the tool for such type of works and highlight the importance of developing and improving tools of this kind, fostering a synergistic collaboration between software developers, artificial intelligence experts, and researchers working on systematic reviews.

The significant heterogeneity observed across studies suggests that not all products within each food group have consistent associations with mortality, highlighting the necessity for more detailed analyses of subcategories. In terms of the quality of the included meta-analyses, the most common shortcoming was not reporting funding of included studies with inherent risk of conflicts of interests in the included cohorts and lack of protocols with risks of arbitrary choices, for example on thresholds for study inclusion and exclusions. Furthermore, even though we only selected articles published in English, we did not identify relevant articles in other languages based on screening of abstracts and titles.

In conclusion, a diet rich in nuts, whole grains, fruits, vegetables, and fish is associated with reduced all-cause mortality, indicating that these food groups play a protective role in overall health and longevity. Comparisons between high and low consumption levels suggest associations for increased intake of legumes and white meat and reduced risk of mortality, however with slightly more uncertainty. However, higher consumption of red and processed meats as well as sugar-sweetened beverages is associated with an increased mortality risk, supporting limiting intakes of these for the sake of longevity. A high intake of added sugars, refined grains and eggs also shows a non-significant trend to increased mortality. No clear associations with mortality were found for dairy products. These conclusions are based on observational evidence from >1 million participants across numerous studies for many of the food groups, mostly adjusted for a range of potential confounders. However, substantial heterogeneity was observed across the studies, potentially arising from differences in the profile of food products eaten within each category and differences in mortality risk. This variability highlights the complexity of dietary patterns and their health impacts, emphasizing the need for further research that explores subgroup differences within food groups. Such efforts could clarify whether certain types of fruits, vegetables, meats, or grains are more or less beneficial, and how factors like preparation methods, dietary context, and cultural influences contribute to health outcomes.

## Author contributions

The authors’ responsibilities were as follows – all authors: contributed to conceiving and designing the study; ATO, RB, MA: led screening and data extractions; RB, MP, ATO, LTF: conducted the analysis; LTF: supervised the study; ATO, RB, LTF: drafted the first manuscript; and all authors: read and approved the final manuscript, have full access to the data, and take responsibility for the integrity of the data and the accuracy of the data analysis.

## Data availability

Data are available in supplementary data files.

## Funding

The authors were funded by their respective institutions. ATO, EJA, and LMT were funded by Western Norway Regional Health Authority (“Strategiske forskningsmidler” through the ATLAS4LAR-project). RB was funded by the Foundation Dam (grant number SDAM_FOR462258) in collaboration with the patient organization LHL and co-funding from the University of Bergen. The funders had no role in study design, data collection and analysis, decision to publish, or preparation of the manuscript. MA and AL were funded with the co-financing of the Italian Ministry of University and Research in the framework of PNC “DARE – Digital lifelong prevention project” (PNC0000002 – CUP B53C22006450001). The views and opinions expressed are solely those of the authors and do not necessarily reflect those of the European Union, nor can the European Union be held responsible for them.

## Conflict of interest

The authors report no conflicts of interest.
