# Supplementary material for: Umbrella Review of Systematic Reviews and Meta-analyses on Consumption of Different Food Groups and Risk of All-cause Mortality
Source: Adv Nutr. 2025 Feb 15;16(4):100393. doi: 10.1016/j.advnut.2025.100393 (PMC11931306; doi:10.1016/j.advnut.2025.100393)

**Appendix:** Overview of supplementary tables and figures

1. Supplementary Text 1: Search string
2. Supplementary Text 2: ASReview Description
3. Supplementary Text 3: Non-linear dose response association between different food groups and All-cause mortality

**Supplementary Text 1: Search string**

Search strategies 19 Nov 2024 – Food groups and life expectancy

**Ovid MEDLINE(R) Epub Ahead of Print and In-Process, In-Data-Review & Other Non-Indexed Citations and Daily <November 18, 2024>; Search date 19 Nov 2024**

1 food/ or bread/ or exp dairy products/ or exp dietary carbohydrates/ or exp dietary fats/ or exp dietary proteins/ or exp eggs/ or flour/ or food, processed/ or fruit/ or exp meat/ or molasses/ or nuts/ or seeds/ or exp edible grain/ or exp vegetables/ 640586

2 (food group* or bread or grain* or cereal* or dairy product* or milk or cheese or yogurt or yoghurt or dietary carbohydrate* or dietary fat* or oil or oils or dietary proteins or egg or eggs or flour or processed food or fruit or berry or berries or citrus or meat or fish or sea food* or chicken or molasses or sugar or sugars or sugar-sweetened beverages or nut or nuts or seed or seeds or vegetable* or legumes or peas or soy or pulses or beans or lentils).ti,ab,kf. 1468065

3 1 or 2 1771040

4 exp Life Expectancy/ or mortality/ or "cause of death"/ or fatal outcome/ or longevity/ 227640

5 (life expectancy or life years or "length of life" or life span* or longevity or mortality or "cause of death" or fatal outcome* or "all cause mortality" or "years of potential life lost").ti,ab,kf. 1262939

6 4 or 5 1381210

7 eating/ or drinking/ or food preferences/ 86831

8 (intake* or consum* or eat* or diet*).ti,ab,kf. 1524833

9 7 or 8 1550697

10 3 and 6 and 9 15959

11 meta-analysis/ or "systematic review"/ 369612

12 ((systematic* adj3 (review* or overview*)) or (integrative adj3 (review* or overview*)) or (collaborative adj3 (review* or overview*)) or (meta analy* or metanaly* or meta-analy* or metaanaly* or systematic review*)).ti,ab,kf. 535370

13 11 or 12 565374

14 10 and 13 1003

<https://ovidsp.ovid.com/ovidweb.cgi?T=JS&NEWS=N&PAGE=main&SHAREDSEARCHID=7XgPd4w996bnQFSxZAHyrB3aCvjvYL4zmMHVJxEwvygLa2Enk0vqYljI4m7iUHEv7>

Comments on OVID-databases:

/ = search on subject heading

Exp = search on a subject heading, expanded to include narrower terms

Ti,ab,kf = search in title, abstract and authors keywords of the article

Adjn = The adjacency operator (ADJn) retrieves records that contain search terms within a specified number 
(*n-1*) of words from each other in any order

**Embase (Ovid) <1974 to 2024 November 18>; Search date 19 Nov 2024**

1 food/ or exp bakery product/ or exp bran/ or exp dairy product/ or exp edible oil/ or exp egg/ or fat/ or exp flour/ or exp food grain/ or exp fruit/ or margarine/ or exp meat/ or exp noodle/ or exp nut/ or pasta/ or exp poultry product/ or exp processed food/ or exp sea food/ or exp soy food/ or exp vegetable/ or exp whole food/ 919744

2 (food group* or bread or grain* or cereal* or dairy product* or milk or cheese or yogurt or yoghurt or dietary carbohydrate* or dietary fat* or oil or oils or dietary proteins or egg or eggs or flour or processed food or fruit or berry or berries or citrus or meat or fish or sea food* or chicken or molasses or sugar or sugars or sugar-sweetened beverages or nut or nuts or seed or seeds or vegetable* or legumes or peas or soy or pulses or beans or lentils).ti,ab,kf. 1616504

3 1 or 2 2049449

4 food intake/ or drinking/ or eating/ or fish consumption/ or fruit consumption/ or meat consumption/ or vegetable consumption/ 236889

5 (intake* or consum* or eat* or diet*).ti,ab,kf. 1927349

6 4 or 5 1984173

7 exp life expectancy/ or mortality/ or all cause mortality/ or "years of potential life lost"/ or longevity/ 1115585

8 (life expectancy or life years or "length of life" or life span* or longevity or mortality or "cause of death" or fatal outcome* or "all cause mortality" or "years of potential life lost").ti,ab,kf. 1849028

9 7 or 8 2157379

10 3 and 6 and 9 21829

11 exp meta analysis/ or "systematic review"/ 637300

12 ((systematic* adj3 (review* or overview*)) or (integrative adj3 (review* or overview*)) or (collaborative adj3 (review* or overview*)) or (meta analy* or metanaly* or meta-analy* or metaanaly* or systematic review*)).ti,ab,kf. 664125

13 11 or 12 813961

14 10 and 13 1433

<https://ovidsp.ovid.com/ovidweb.cgi?T=JS&NEWS=N&PAGE=main&SHAREDSEARCHID=2534MmDPS466tFKOYqXdiBgrWGXj2uHtVrph4fasJfkdfuBOUJY4vJX19jeVaydTP>

**Epistemonikos (**[Epistemonikos foundation](http://foundation.epistemonikos.org/)); **Search date 19 Nov 2024**

Title/abstract-searches:
("food group" or "food groups" or bread or grain* or cereal* or "dairy product" or "dairy products" or milk or cheese or yogurt or yoghurt or "dietary carbohydrate*" or "dietary fat*" or oil or oils or "dietary proteins" or egg or eggs or flour or "processed food" or fruit or berry or berries or citrus or meat or fish or sea food* or chicken or molasses or sugar or sugars or "sugar-sweetened beverages" or nut or nuts or seed or seeds or vegetable* or legumes or peas or soy or pulses or beans or lentils)

AND (intake* or consum* or eat* or diet*)

AND ("life expectancy" or "life years" or "length of life" or "life span" or longevity or mortality or "cause of death" or "fatal outcome" or "fatal outcomes" or "all cause mortality" or "years of potential life lost")

AND ("systematic review" or "systematic overview" or "integrative review" or "integrative overview" or "integrative literature review" or "integrative literature overview" or "collaborative review" or "collaborative overview" or "meta analysis" or metanaly* or meta-analy* or metaanaly*)
= 730 results

(1 AND 2 AND 3) + Filter in Epistemonikos for systematic reviews (alternative search for SR): 810 results
(combined with 730 results = 885)

(1 AND 2 AND 3) + Filter in Epistemonikos used for Broad synthesis: 81

**Web of Science (Clarivate), WOS.SCI: 1945 to 2024, WOS.AHCI: 1975 to 2024, WOS.ESCI: 2019 to 2024, WOS.SSCI: 1956 to 2024; Search date 19 Nov 2024**

1: TS=((("food group*" or bread or grain* or cereal* or "dairy product*" or milk or cheese or yogurt or yoghurt or "dietary carbohydrate*" or "dietary fat*" or oil or oils) NEAR/2 (intake* or consum* or eat* or diet*))) Results: 81654

2: TS=((("dietary proteins" or egg or eggs or flour or "processed food" or fruit or berry or berries or citrus or meat or fish or "sea food*" or chicken or molasses) NEAR/2 (intake* or consum* or eat* or diet*))) Results: 94566

3: TS=(((sugar or sugars or "sugar-sweetened beverages" or nut or nuts or seed or seeds or vegetable* or legumes or peas or soy or pulses or beans or lentils) NEAR/2 (intake* or consum* or eat* or diet*))) Results: 56862

4: #1 OR #2 OR #3 Results: 202161

5: TS=(("life expectancy" or "life years" or "length of life" or "life span" or longevity or mortality or "cause of death" or "fatal outcome" or "fatal outcomes" or "all cause mortality" or "years of potential life lost")) Results: 1641524

6: (TI=(((systematic* NEAR/2 (review* or overview*)) or (integrative NEAR/2 (review* or overview*)) or (collaborative NEAR/2 (review* or overview*)) or ("meta analy*" or metanaly* or meta-analy* or metaanaly* or "systematic review*")))) OR (AB=(((systematic* NEAR/2 (review* or overview*)) or (integrative NEAR/2 (review* or overview*)) or (collaborative NEAR/2 (review* or overview*)) or ("meta analy*" or metanaly* or meta-analy* or metaanaly* or "systematic review*")))) Results: 633122

7: #4 AND #5 AND #6 Results: 818

<https://www.webofscience.com/wos/woscc/summary/15f4a61a-1115-40b5-bd07-b15103fe4d13-0128955541/relevance/1>

Comments:
Web of Science does not use subject headings for indexing. Therefore, we used proximity operators and specific search fields for free text word searches, for reducing the number of irrelevant hits.
Line 1-3: free text word searches for food groups and intake are combined with proximity operator. This means words from both elements must be close to each other with up to 2 words between.
Line 6: Study design was searched in title or abstract, as in the other databases.

TS = Topic search = search in title, abstract and author key words

TI = search in title

AB = Search in abstract

Near/2 = The adjacency operator (NEAR/n) retrieves records that contain search terms within a specified number (n) of words between the search terms in any order

**Supplementary Text 2: Simulated screening with ASReview**

Aim

In order to evaluate the potential benefits resulting from the semi-automatization of the process of titles and abstracts screenings, a set of simulation of such task was run using the software ASReview. This allowed to assess the amount of time potentially saved using different settings of the software in a similar situation, as well as the potential loss in terms of screening sensitivity.

Methods and data

AsReview, an NLP-based tool to streamline titles and abstracts screening

The screening simulations were performed using ASReview v1.5 (ASReview LAB developers, 2024). ASReview is a tool which exploits active learning and natural language processing (NLP) to streamline the process of titles and abstracts screening. More specifically, it learns the features of the relevant articles from the decision (inclusion/exclusion) of the human reviewers, and, based on that, it iteratively suggests to the reviewer which article to screen next, so allowing to speed up the screening process (van de Schoot et al., 2021). In this way, most of the relevant papers are identified by the reviewer in the first part of the process, reducing the time needed to identify all or almost all the papers satisfying the inclusion criteria, and saving the reviewers from the effort of going through the whole corpus of retrieved studies, which is usually very noisy.

In order to work, ASReview needs the user to set a “prior knowledge”, which consists in labelling at least one of the candidate papers as relevant and one as irrelevant before even starting screening. Another important feature of ASReview is that it allows the screener to set a stopping rule, leaving them in control of how to set the tradeoff between screening sensitivity and time saving. Two examples of stopping rules are: “100 screened irrelevant papers in a row” and “<10 relevant papers across 100 papers screened”; the former is more sensitivity-oriented, while the latter is more time-saving-oriented.

In this study, after carrying out the screening in the conventional way, we used the “Simulation” function of ASReview. This function allows to simulate an already performed screening in order to find out how it would have evolved if ASReview had been used as a supporting tool. More specifically, it allows to define how long it would have taken to identify each relevant paper and, ultimately, how long it would have taken to identify *all* the relevant papers. Based on this, one can estimate how much time would have been saved with each stopping rule and how much it would have “costed” in terms of non-retrieved relevant papers.

Alongside the stopping rule, ASReview also allows to select different options for the classifier, the feature extractor, the query model, and the balance strategy. The simulations were finalized at identifying the optimal combination of all these settings for the set of candidate articles and the inclusion criteria of the current review, so to extract an evidence-based suggestion for future similar studies.

Data

The simulations were performed on three data sets, one for each reviewer, including the title and abstract of each candidate article, as well as the decision of each reviewer on that article (inclusion/exclusion) *before* the possible conflict discussion. All the screeners screened the whole set of candidate papers: AO labelled 83 papers as relevant, 54 of which were actually considered relevant after conflicts discussion (“true relevant”, TR) and 29 of which were not considered relevant after conflicts discussion (“false relevant”, FR). MA labelled 94 papers as relevant, .. of which were TR and .. were FR. RB labelled 96 papers as relevant, with 49 TR and 47 FR.

Simulations design

To accomplish the aim, a four-step process was performed, carrying out several simulations on the 3 data sets of decisions of the reviewers.

In each step, the following metrics were used to evaluate the performance of the possible settings:

- Screening effort saved: percentage of papers not screened yet when the stopping rule was reached;
- Recall: percentage of papers considered relevant by the screener (before conflict discussion) and already identified when the stopping rule was reached;
- True relevant missed: number of papers considered relevant after conflict discussion and not yet identified when the stopping rule was reached (i.e., useless conflicts generated);
- False relevant missed: number of papers considered relevant by the screener but considered irrelevant after conflicts discussion, and not yet identified when the stopping rule was reached (i.e., useless conflicts avoided);
- Simulation time: actual time-machine needed to perform the simulation, i.e., to extract the features and to provide feedback.

First assessment

The first explorative assessment consisted in performing one simulation with each possible combination of settings, in order to have a first understanding of which were good candidates settings. At this stage, the data referring to RB were used, as they present the median value of relevant papers.

The paper “Food groups and risk of all-cause mortality: a systematic review and meta-analysis of prospective studies” (Schwingshackl et al., 2017) was selected as relevant prior knowledge, while a random paper was selected as irrelevant prior knowledge (as suggested by the ASReview documentation).

All the possible combinations of options, for a total of 96, were used. The candidate options were the following:

- Classifiers: Naïve Bayes, Logistic regression, Random forest, Support vector machine
- Feature extraction methods: TF-IDF, LSTMEmbedding
- Query strategies: Cluster query, Max query, Max random query, Max uncertainty query
- Balance strategies: Double, Simple, Undersampling

The parameter update was set at each 10 papers screened, and the stopping rule was set as “100 irrelevant papers in a row”. More information about the meaning of these settings can be found in the documentation of ASReview (www.asreview.nl).

At this stage, a setting was considered “good” if it complied with the following criteria:

- Screening effort saved: >= 50%
- Recall: >=80%
- True relevant missed: 0

All the “good settings” were kept for the subsequent testing stages.

Second assessment

In the second stage, the settings which showed “good” performance in the previous round were used again to perform simulations on the data referring to AO and MA. The prior knowledge and the stopping rule were set as in the first stage.

At this point, the average performance of each setting across the three data sets were computed, in order to perform an overall assessment. Based on this, the best setting was selected.

Third assessment

In the third stage, a set of candidate stopping rules were tested with the best setting resulting from the previous stage, with the aim of finding the best performing one.

The candidate stopping rules were: 25, 50, or 100 irrelevant papers in a row. 10 simulations were run for each combination of stopping rule and data set, setting as prior relevant paper the same paper as the previous stages, and randomly setting the prior irrelevant paper at each simulation.

Fourth assessment

The last stage consisted of a final simulation conducted on the three data sets using the best performing setting and stopping rule. This allowed to give a closer look at the results of the simulated screening, and to estimate the performance of the optimal setting not only in terms of recall, but also counting the actual estimated number of screenings avoided and conflicts avoided for each screener.

The included prior was the same as the previous stages, while the excluded prior, randomly set, was “Fermented Dairy Products, Probiotic Supplementation, and Cardiometabolic Diseases: A Systematic Review and Meta-analysis” (Companys et al., 2020).

Results

First assessment

The first stage of assessment yielded 15 “good settings”: Table 1 shows in detail their definition and performance. The percentage of screening effort saved by these savings ranged between 52.11% and 68.04%; the recall ranged between 80.21% and 97.92%. It is very noteworthy that the setting 08 yielded a perfect recall on “true relevant” records (49/49), and a quite low recall on “false relevant” records (28/47). This means that using ASReview with this setting could have prevented RB to raise almost half of the conflicts on irrelevant papers that he was involved in.

| **ID** | **Classifier** | **Feat ext** | **Query**  **model** | **Balance strategy** | **Sim time (s)** | **Screening**  **time**  **saved (%)** | **Recall** | **TR found** | **FR found** | **TR misssed** | **FR avoided** |
| --- | --- | --- | --- | --- | --- | --- | --- | --- | --- | --- | --- |
| RB000 | logistic | tfidf | Max | double | 8.89 | 54.50 | 0.98 | 49 | 45 | 0 | 2 |
| RB001 | logistic | tfidf | Max | simple | 9.48 | 58.32 | 0.98 | 49 | 45 | 0 | 2 |
| RB002 | logistic | tfidf | Max | under | 5.46 | 68.04 | 0.94 | 49 | 41 | 0 | 6 |
| RB003 | logistic | tfidf | MaxUncert | double | 11.29 | 60.38 | 0.97 | 49 | 44 | 0 | 3 |
| RB004 | logistic | tfidf | MaxUncert | simple | 13.46 | 57.77 | 0.98 | 49 | 45 | 0 | 2 |
| RB005 | logistic | tfidf | MaxUncert | under | 8.02 | 58.49 | 0.97 | 49 | 44 | 0 | 3 |
| RB006 | nb | tfidf | Max | double | 3.49 | 54.38 | 0.96 | 49 | 43 | 0 | 4 |
| RB007 | nb | tfidf | MaxRand | double | 6.26 | 67.81 | 0.80 | 49 | 28 | 0 | 19 |
| RB008 | rf | tfidf | Max | double | 124.12 | 52.11 | 0.96 | 49 | 43 | 0 | 4 |
| RB009 | rf | tfidf | MaxUncert | double | 125.97 | 60.88 | 0.92 | 49 | 39 | 0 | 8 |
| RB010 | rf | tfidf | MaxUncert | under | 72.37 | 52.28 | 0.95 | 49 | 42 | 0 | 5 |
| RB011 | svm | tfidf | Max | double | 449.31 | 59.27 | 0.96 | 49 | 43 | 0 | 4 |
| RB012 | svm | tfidf | Max | under | 106.43 | 63.21 | 0.96 | 49 | 43 | 0 | 4 |
| RB013 | svm | tfidf | MaxUncert | double | 456.82 | 62.60 | 0.96 | 49 | 43 | 0 | 4 |
| RB014 | svm | tfidf | MaxUncert | under | 104.26 | 57.66 | 0.96 | 49 | 43 | 0 | 4 |

Table 1: Summary of the performance of the “good settings” identified during the first round of simulations on RB data. TR: True relevant (i.e., considered relevant by RB and confirmed after conflicts discussion); FR: False relevant (i.e., considered relevant by RB, but discarded after conflicts discussion).

Second assessment

The simulated performance of AO and MA with the 15 good settings are reported in Table 2 and 3, respectively.

For AO, the effort saved ranged between 57.82% and 79.25%, with recall ranging from 95.18% to 98.80%. It is noteworthy that all the simulations yielded to 1 or 2 TR missing, while only three settings yielded to any FR avoided, with a maximum of 2 (ID 007 and 010).

| **ID** | **Sim time (s)** | **Scr time saved (%)** | **Recall** | **TP** | **FP** | **TP miss** | **FP avoid** |
| --- | --- | --- | --- | --- | --- | --- | --- |
| AO000 | 6.42 | 78.69 | 0.99 | 53 | 29 | 1 | 0 |
| AO001 | 10.84 | 79.19 | 0.99 | 53 | 29 | 1 | 0 |
| AO002 | 5.33 | 76.30 | 0.99 | 53 | 29 | 1 | 0 |
| AO003 | 11.12 | 73.92 | 0.99 | 53 | 29 | 1 | 0 |
| AO004 | 12.01 | 79.25 | 0.99 | 53 | 29 | 1 | 0 |
| AO005 | 8.10 | 77.75 | 0.99 | 53 | 29 | 1 | 0 |
| AO006 | 2.84 | 72.92 | 0.99 | 53 | 29 | 1 | 0 |
| AO007 | 5.27 | 57.82 | 0.95 | 52 | 27 | 2 | 2 |
| AO008 | 116.6 | 66.20 | 0.99 | 53 | 29 | 1 | 0 |
| AO009 | 115.42 | 64.32 | 0.98 | 53 | 28 | 1 | 1 |
| AO010 | 65.87 | 70.31 | 0.96 | 53 | 27 | 1 | 2 |
| AO011 | 326.52 | 73.58 | 0.99 | 53 | 29 | 1 | 0 |
| AO012 | 86.44 | 74.58 | 0.99 | 53 | 29 | 1 | 0 |
| AO013 | 330.73 | 78.25 | 0.99 | 53 | 29 | 1 | 0 |
| AO014 | 89.79 | 76.53 | 0.99 | 53 | 29 | 1 | 0 |

Table 2: Summary of the performance of the “good settings” on the AO data. TR: True relevant (i.e., considered relevant by AO and confirmed after conflicts discussion); FR: False relevant (i.e., considered relevant by AO, but discarded after conflicts discussion).

For MA, there was one setting (ID 012) yielding very poor results, with the stopping rule reached very soon and only 2 relevant articles identified. Another setting (ID 007) yielded quite poor results, with 65% recall and 5 TR missing. Among the other settings, the effort saved ranged between 36.13% and 68.04%, with recall ranging from 95.18% to 98.80%. 4 settings yielded no missing TR, and all the others presented 1 missing TR; all the settings yielded to some level of FR avoidance, with a maximum of 16 (ID 009).

| **ID** | **Sim time (s)** | **Scr time saved (%)** | **Recall** | **TP** | **FP** | **TP miss** | **FP avoid** |
| --- | --- | --- | --- | --- | --- | --- | --- |
| MA000 | 6.58 | 68.04 | 0.87 | 62 | 34 | 1 | 13 |
| MA001 | 8.23 | 40.62 | 0.97 | 63 | 44 | 0 | 3 |
| MA002 | 5.58 | 44.84 | 0.97 | 63 | 44 | 0 | 3 |
| MA003 | 11.55 | 47.17 | 0.97 | 63 | 44 | 0 | 3 |
| MA004 | 11.9 | 56.71 | 0.9 | 62 | 37 | 1 | 10 |
| MA005 | 8.17 | 67.15 | 0.88 | 62 | 35 | 1 | 12 |
| MA006 | 3.91 | 48.89 | 0.95 | 63 | 41 | 0 | 6 |
| MA007 | 4.46 | 70.81 | 0.65 | 58 | 14 | 5 | 33 |
| MA008 | 123.6 | 36.13 | 0.95 | 62 | 42 | 1 | 5 |
| MA009 | 125.57 | 58.05 | 0.85 | 62 | 31 | 1 | 16 |
| MA010 | 73.11 | 37.68 | 0.92 | 62 | 39 | 1 | 8 |
| MA011 | 543.99 | 57.16 | 0.94 | 62 | 41 | 1 | 6 |
| MA012 | 99.65 | 90.23 | 0.02 | 2 | 0 | 61 | 47 |
| MA013 | 520.67 | 53.72 | 0.94 | 62 | 41 | 1 | 6 |
| MA014 | 113.98 | 52.89 | 0.93 | 62 | 40 | 1 | 7 |

Table 3: Summary of the performance of the “good settings” on the MA data. TR: True relevant (i.e., considered relevant by MA and confirmed after conflicts discussion); FR: False relevant (i.e., considered relevant by MA, but discarded after conflicts discussion).

Table 4 shows the average metrics observed for each setting across the simulations described above. The settings 001, 003, and 002 attained the best recall; the same settings, as well as setting 006, obtained the best recall of TR articles (i.e., spotting articles relevant also after conflicts discussion); 009 and 010 reached the highest FR avoidance rate (i.e., avoiding useless conflict discussions); 005, 000 and 013 were the fastest in reaching the stopping rule.

| **ID** | **Simulation time** | **Recall** | **TR recall** | **FR avoid rate** | **Screening effort saved (%)** |
| --- | --- | --- | --- | --- | --- |
| 000 | 7.29 | 94.66 | 98.85 | 10.64 | 67.08 |
| 001 | 9.51 | 98.00 | 99.38 | 3.55 | 59.38 |
| **002** | **5.45** | **96.61** | **99.38** | **6.38** | **63.06** |
| 003 | 11.32 | 97.65 | 99.38 | 4.25 | 60.49 |
| 004 | 12.46 | 95.57 | 98.85 | 8.51 | 64.58 |
| 005 | 8.10 | 94.62 | 98.85 | 10.64 | 67.80 |
| 006 | 3.41 | 96.39 | 99.38 | 7.09 | 58.73 |
| 008 | 121.44 | 96.39 | 98.85 | 6.38 | 51.48 |
| 009 | 122.32 | 91.27 | 98.85 | 18.17 | 61.08 |
| 010 | 70.45 | 94.33 | 98.85 | 11.52 | 53.42 |
| 011 | 439.94 | 96.09 | 98.85 | 7.09 | 63.34 |
| 013 | 436.07 | 96.09 | 98.85 | 7.09 | 64.86 |
| 014 | 102.67 | 95.79 | 98.85 | 7.80 | 62.36 |

Table 4: Summary of the average performance of the “good settings” across all the data. TR: True relevant; FR: False relevant. Settings 007 and 012 were excluded because of the poor performance shown with the MA data.

Summarizing, it is possible to notice what follows:

- Most of the best-performing settings used the logistic regression as classifier (IDs 000, 001, 002, 003, 005). Among those, the setting 002 looked like a very good tradeoff between sensitivity and specificity, as it yielded the best TR rate and slightly higher FR avoidance rate and time saved compared to the other settings.
- The settings with random forest as classifiers tended to show a lower recall compared with the others, but it was mostly due to a better specificity, i.e., a higher FR avoidance rate (IDs 009 and 010).
- The settings with naïve bayes and support vector machine as classifiers did not obtain any particularly significant performance compared to the other ones, and in particular the settings with SVM had a longer training time (i.e., simulation time).

Based on this, and trying to go for a slightly conservative choice (i.e., better sensitivity, even without perfect sensitivity), setting 02 was considered the best tradeoff between all the assessed characteristics. It is defined as follows:

- Classifier: Logistic regression
- Feature extractor: TfIdf
- Query model: MaxQuery
- Balance strategy: Undersampling

Such setting was selected as the optimal one and further tested.

Third assessment

The results of the simulations with different stopping rules are summarized in Table 5. As expected, as the stopping rule gets less strict (i.e., the number of irrelevant papers in a row needed decreases), the amount of time saved increases and the recall decreases. It is noteworthy that, especially when going from 50 to 100 irrelevant papers in a row, the loss in terms of recall is almost totally due to the increased FR avoidance. Based on this, “*50 irrelevant papers in a row*” was chosen as a stopping rule. It provided on average a high overall recall (>89%) and TR recall (almost 98%), with a substantial amount of screening effort saved (over 75%).

| **Stopping rule** | **Recall** | **TR recall** | **FR avoid rate** | **Time saved (%)** |
| --- | --- | --- | --- | --- |
| 25 | 79.31 | 94.58 | 39.73 | 84.20 |
| **50** | **89.45** | **97.89** | **20.57** | **75.76** |
| 100 | 96.2 | 99.12 | 7.38 | 62.90 |

Table 5: Average results of the simulations with different stopping rules.

Fourth assessment

Figure 1 shows the recall plots of the last round of simulated screenings. More specifically, Figure 1a shows that AO would have found almost all the same relevant papers before the stopping rule would verify. Conversely, Figure 1b and 1c, show that MA and RB would have missed some of the papers that they considered relevant, in case they had used ASReview.


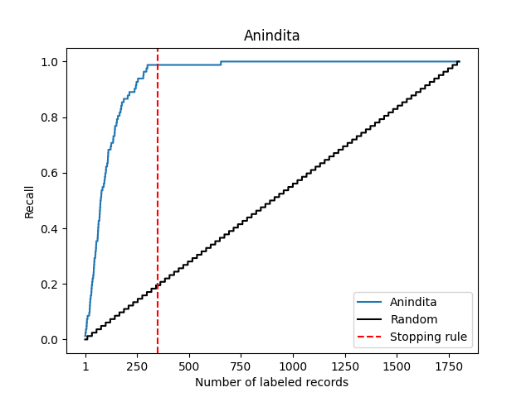

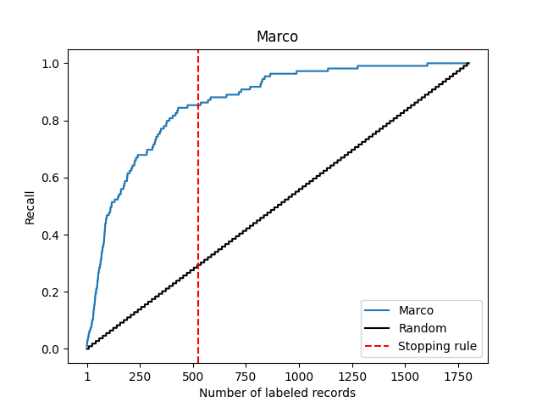

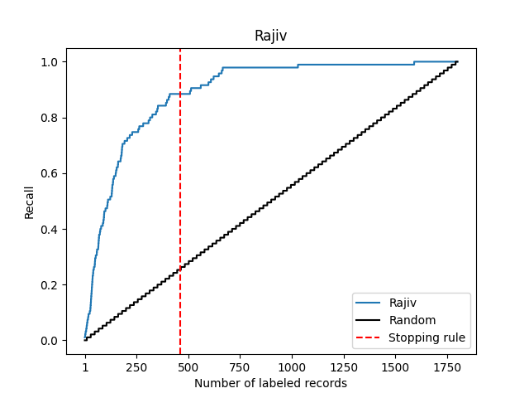


Figure 1: Recall plots of the simulated screenings of AO (a), MA (b), and RB (c).

The aggregated results reported in Table 6 show that most of the relevant records missed in the simulated screenings were actually “false relevant” (15 for MA, 10 for RB, 0 for AO).

|  | **AO** | **MA** | **RB** |
| --- | --- | --- | --- |
| **Records screened** | 351 | 525 | 461 |
| **N. marked relevant** | 82 | 94 | 85 |
| **TR found** | 53 | 62 | 48 |
| **FR found** | 29 | 32 | 37 |
| **TR missed** | 1* | 1* | 1* |
| **FR missed** | 0 | 15 | 10 |

Table 6: Results of the last simulations on the three data sets. *True relevant discarded in the real screening after further assessment.

A closer inspection of the results of each simulation showed that 49 articles were considered relevant by all the screeners in the simulation, against the 50 that were considered relevant by all in the actual screening process. The other article was missed by all the screeners in the simulations: this means that, using ASReview with the selected setting, such paper would not have made it to the full text assessment. Despite this is in principle an undesired behavior, that specific paper (Fadnes et al., 2022) was actually excluded from the pool of candidates before the full text screening subsequent to further internal discussions among the screeners and the other co-authors. This means that also the *apparent* loss of information due to the use of ASReview would have actually resulted in a gain in terms of specificity.

Moreover, 88 records were considered relevant by one or two screeners in the simulations, against 108 in the real screening. This means that at least 20 useless conflicts would have been avoided if ASReview had been used.

Discussion and conclusions

The results of the performed simulations showed the robustness of ASReview in this specific context of application. In particular, 15 potentially “good” settings of the software were assessed. Almost all of them showed good recall, conflicts avoidance and time saving capacity. After performing several tests, a logistic regression with TF-IDF, MaxQuery and Undersampling were selected as the most reliable and best performing settings. This was associated with a stopping rule of “50 irrelevant papers in a row”.

The final simulation allowed to estimate precisely what would have happened if ASReview had been used for the screening in the present study. The simulations showed that all the screeners would have saved around 75% of their screening time *without* missing any relevant records. Moreover, the use of ASReview would have reduced the number of mistakenly included records, resulting in the avoidance of at least 20 useless conflicts and 1 incorrect inclusion.

These results are coherent with the existing evidence about the robustness and reliability of ASReview, and they provide a further assurance about the possibility of using the tool to streamline the screening process of umbrella reviews similar to the present one, without reasonable suspicion of information loss.

References

ASReview LAB developers. (2024). *ASReview LAB - A tool for AI-assisted systematic reviews (v1.5)*.

Companys, J., Pla-Pagà, L., Calderón-Pérez, L., Llauradó, E., Solà, R., Pedret, A., & Valls, R. M. (2020). Fermented Dairy Products, Probiotic Supplementation, and Cardiometabolic Diseases: A Systematic Review and Meta-analysis. *Advances in Nutrition*, *11*(4), 834–863. https://doi.org/10.1093/advances/nmaa030

Fadnes, L. T., Økland, J.-M., Haaland, Ø. A., & Johansson, K. A. (2022). Estimating impact of food choices on life expectancy: A modeling study. *PLOS Medicine*, *19*(2), e1003889. https://doi.org/10.1371/journal.pmed.1003889

Schwingshackl, L., Schwedhelm, C., Hoffmann, G., Lampousi, A.-M., Knüppel, S., Iqbal, K., Bechthold, A., Schlesinger, S., & Boeing, H. (2017). Food groups and risk of all-cause mortality: a systematic review and meta-analysis of prospective studies ,. *The American Journal of Clinical Nutrition*, *105*(6), 1462–1473. https://doi.org/10.3945/ajcn.117.153148

van de Schoot, R., de Bruin, J., Schram, R., Zahedi, P., de Boer, J., Weijdema, F., Kramer, B., Huijts, M., Hoogerwerf, M., Ferdinands, G., Harkema, A., Willemsen, J., Ma, Y., Fang, Q., Hindriks, S., Tummers, L., & Oberski, D. L. (2021). An open source machine learning framework for efficient and transparent systematic reviews. *Nature Machine Intelligence*, *3*(2), 125–133. https://doi.org/10.1038/s42256-020-00287-7

**Supplementary Text 3: Non-linear dose response association between different food groups and All-cause mortality**

For added sugars, the dose-response curve showed a J-shaped pattern, with the relative risk above 1 for intakes greater than 10%E, following a linear increasing trend between 10%E and 40%E (23). A J-shaped pattern was also observed for dairy, with RR below 1 for intakes under 400 g/day and mortality associations increasing nearly linearly above 400 g/day (1). For sugar-sweetened beverages, the dose-response curve shows a nearly linear increasing RR across the whole domain of analysis (0 to 2,500ml/day) (9). A similar pattern is seen for processed meat and unprocessed red meats, with a nearly linear pattern in the association with all-cause mortality (1, 10). For refined grains, there also seems to be linear associations in the range 0 to 400g/day (22). For eggs consumption, the dose-response curve is increasing across the whole domain of analysis (0 to 18 eggs per week), with the slope of the curve gradually increasing as the intake increases (44). For fish, the dose-response curve shows a gradual decrease in RR over the whole domain of analysis (0 to 250 g/day) (1), with a steeper slope in the range between 0 and 50 g/day. For legumes, the RR is decreasing linearly across the whole domain of analysis (0 to 160g/day) (1). Nuts show an inverse J-shaped curve, with a decline in RR between 0 and 15g of daily intake, after which plateaus between intakes of 20-30g and then it rises slightly (1). For vegetables, the RR decreases until an intake of around 250g of intake, then plateaus (nonlinearly). The dose-response curve for white meat consumption shows a U-shaped pattern, with a negative slope for low intakes, a plateau between 30 and 40g/day, and a positive slope between 40 and 100g/day (38). Increasing whole grain intake in the range 0 to 220 g/day, shows a non-linear inverse association across the whole spectrum with the steepest decline with from no intakes to about 40g/day (22). Non-linear analysis for fruits and all-cause mortality indicated non-linear inverse association across the whole spectrum from 0-600 g/day reaching maximum inverse associations at intakes around 300 g/day (24).

Note: The references correspond to the manuscript’s referencing number.

**Appendix:** Overview of supplementary figures

1. Supplementary Figure 1: Associations between intake of whole grains for high versus low and all-cause mortality from all meta-analyses HL_Whole grains_ACM
2. Supplementary Figure 2: Associations between intake of whole grains per serving and all-cause mortality from all meta-analyses PS_Whole grains_ACM
3. Supplementary Figure 3: Associations between intake of refined grains for high versus low and all-cause mortality from all meta-analyses HL_Refined grains_ACM
4. Supplementary Figure 4: Associations between intake of refined grains per serving and all-cause mortality from all meta-analyses PS_Refined grains_ACM
5. Supplementary Figure 5: Associations between intake of fruits for high versus low and all-cause mortality from all meta-analyses HL_Fruits_ACM
6. Supplementary Figure 6: Associations between intake of fruits per serving and all-cause mortality from all meta-analyses PS_Fruits_ACM
7. Supplementary Figure 7: Associations between intake of vegetables for high versus low and all-cause mortality from all meta-analyses HL_Vegetables_ACM
8. Supplementary Figure 8: Associations between intake of vegetables per serving and all-cause mortality from all meta-analyses PS_Vegetables_ACM
9. Supplementary Figure 9: Associations between intake of nuts for high versus low and all-cause mortality from all meta-analyses HL_Nuts_ACM
10. Supplementary Figure 10: Associations between intake of nuts per serving and all-cause mortality from all meta-analyses PS_Nuts_ACM
11. Supplementary Figure 11: Associations between intake of legumes for high versus low and all-cause mortality from all meta-analyses HL_Legumes_ACM
12. Supplementary Figure 12: Associations between intake of legumes per serving and all-cause mortality from all meta-analyses PS_Legumes_ACM
13. Supplementary Figure 13: Associations between intake of fish and fish products for high versus low and all-cause mortality from all meta-analyses HL_Fish_ACM
14. Supplementary Figure 14: Associations between intake of fish and fish products per serving and all-cause mortality from all meta-analyses PS_Fish_ACM
15. Supplementary Figure 15: Associations between intake of eggs for high versus low and all-cause mortality from all meta-analyses HL_Eggs_ACM
16. Supplementary Figure 16: Associations between intake of eggs per serving and all-cause mortality from all meta-analyses PS_Eggs_ACM
17. Supplementary Figure 17: Associations between intake of dairy products for high versus low and all-cause mortality from all meta-analyses HL_Dairy_ACM
18. Supplementary Figure 18: Associations between intake of dairy products per serving and all-cause mortality from all meta-analyses PS_Dairy_ACM
19. Supplementary Figure 19: Associations between intake of processed meat for high versus low and all-cause mortality from all meta-analyses HL_Processed meat_ACM
20. Supplementary Figure 20: Associations between intake of processed meat per serving and all-cause mortality from all meta-analyses PS_Processed meat_ACM
21. Supplementary Figure 21: Associations between intake of unprocessed red meat for high versus low and all-cause mortality from all meta-analyses HL_Red meat_ACM
22. Supplementary Figure 22: Associations between intake of unprocessed red meat per serving and all-cause mortality from all meta-analyses PS_Red meat_ACM
23. Supplementary Figure 23: Associations between intake of unprocessed white meat for high versus low and all-cause mortality from all meta-analyses HL_White meat_ACM
24. Supplementary Figure 24: Associations between intake of unprocessed white meat per serving and all-cause mortality from all meta-analyses PS_White meat_ACM
25. Supplementary Figure 25: Associations between intake of sugar-sweetened beverages for high versus low and all-cause mortality from all meta-analyses HL_SSB_ACM
26. Supplementary Figure 26: Associations between intake of sugar-sweetened beverages per serving and all-cause mortality from all meta-analyses PS_SSB_ACM
27. Supplementary Figure 27: Associations between intake of added sugars for high versus low and all-cause mortality from all meta-analyses HL_Added sugars_ACM
28. Supplementary Figure 28: Associations between intake of added sugars per serving and all-cause mortality from all meta-analyses PS_Added sugars_ACM

Supplementary Figure 1: Associations between intake of whole grains for high versus low and all-cause mortality from all meta-analyses HL_Whole grains_ACM


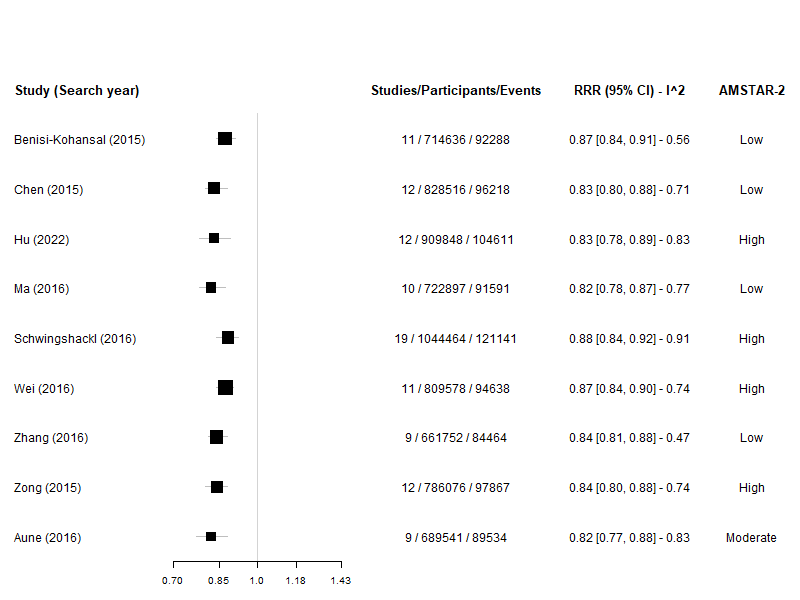


Supplementary Figure 2: Associations between intake of whole grains per serving and all-cause mortality from all meta-analyses PS_Whole grains_ACM


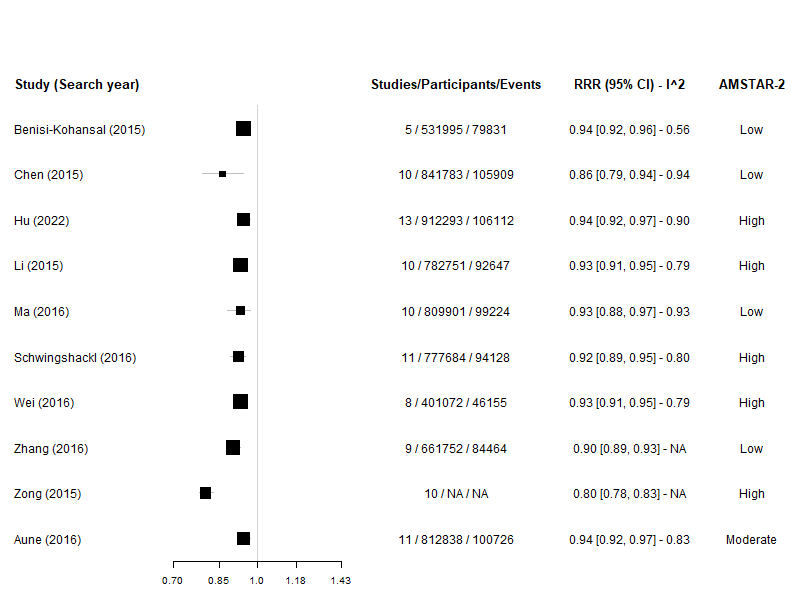


Supplementary Figure 3: Associations between intake of refined grains for high versus low and all-cause mortality from all meta-analyses HL_Refined grains_ACM


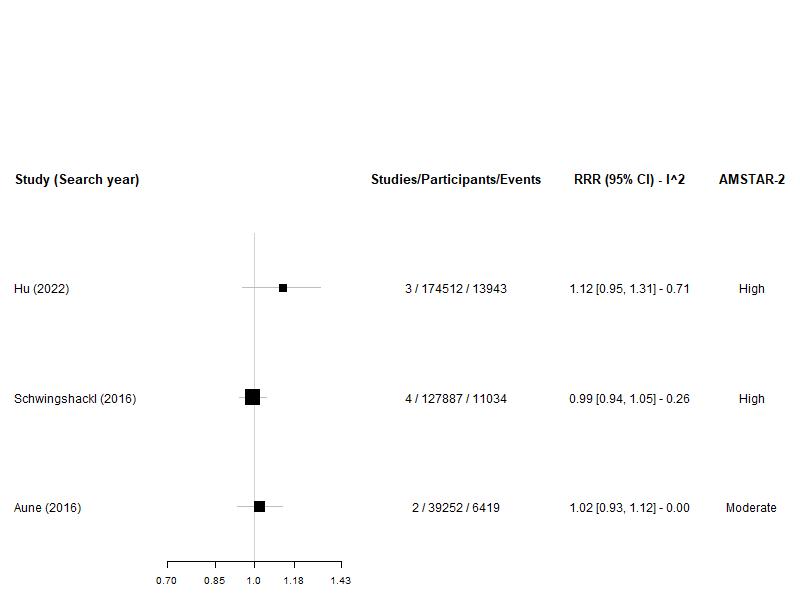


Supplementary Figure 4: Associations between intake of refined grains per serving and all-cause mortality from all meta-analyses PS_Refined grains_ACM


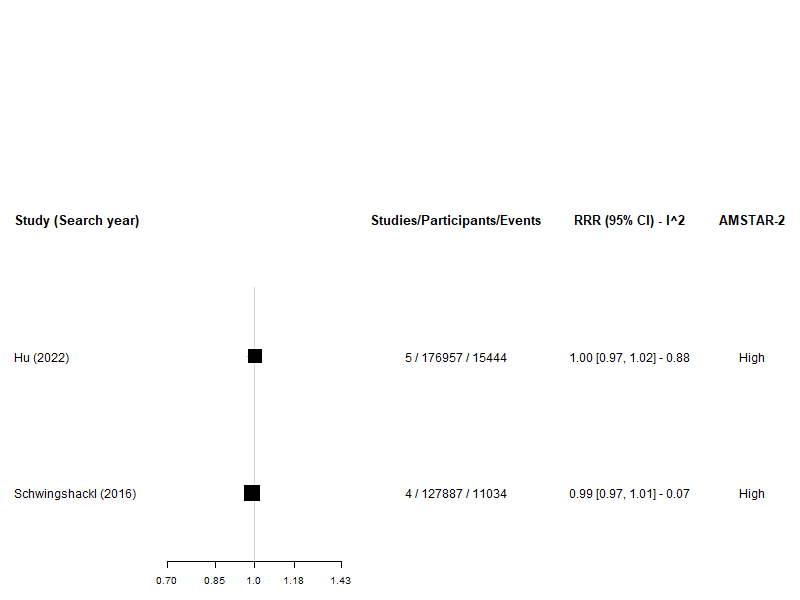


Supplementary Figure 5: Associations between intake of fruits for high versus low and all-cause mortality from all meta-analyses HL_Fruits_ACM


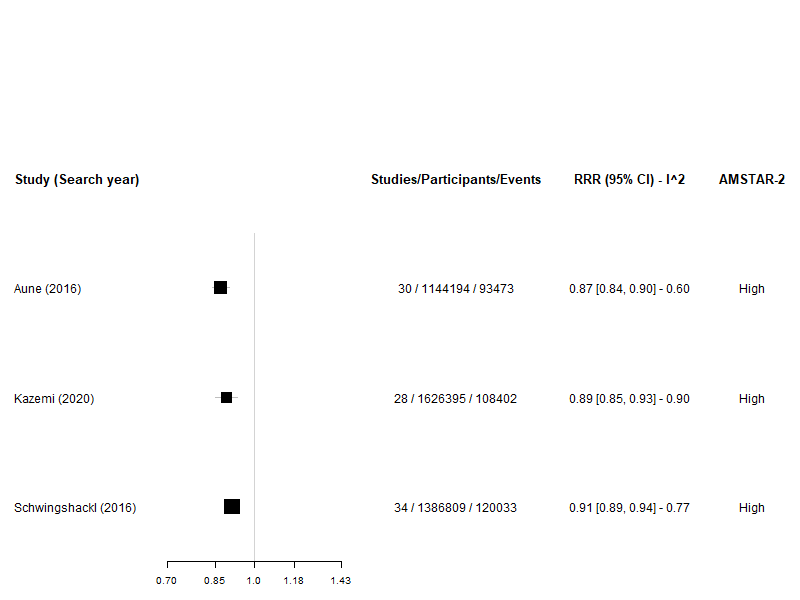


Supplementary Figure 6: Associations between intake of fruits per serving and all-cause mortality from all meta-analyses PS_Fruits_ACM


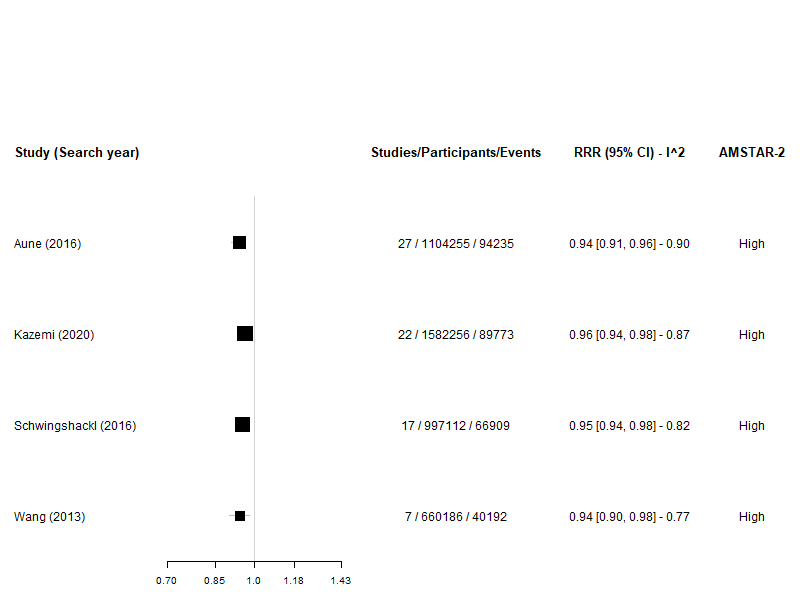


Supplementary Figure 7: Associations between intake of vegetables for high versus low and all-cause mortality from all meta-analyses HL_Vegetables_ACM


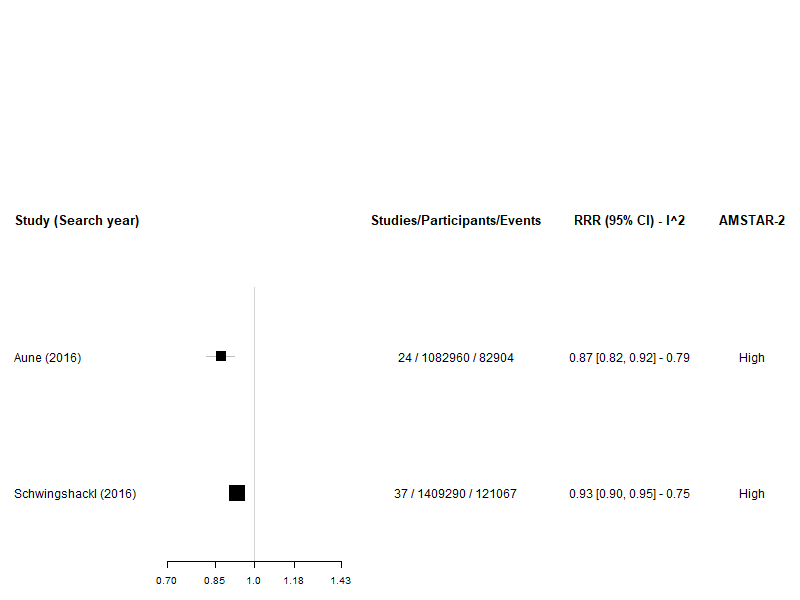


Supplementary Figure 8: Associations between intake of vegetables per serving and all-cause mortality from all meta-analyses PS_Vegetables_ACM


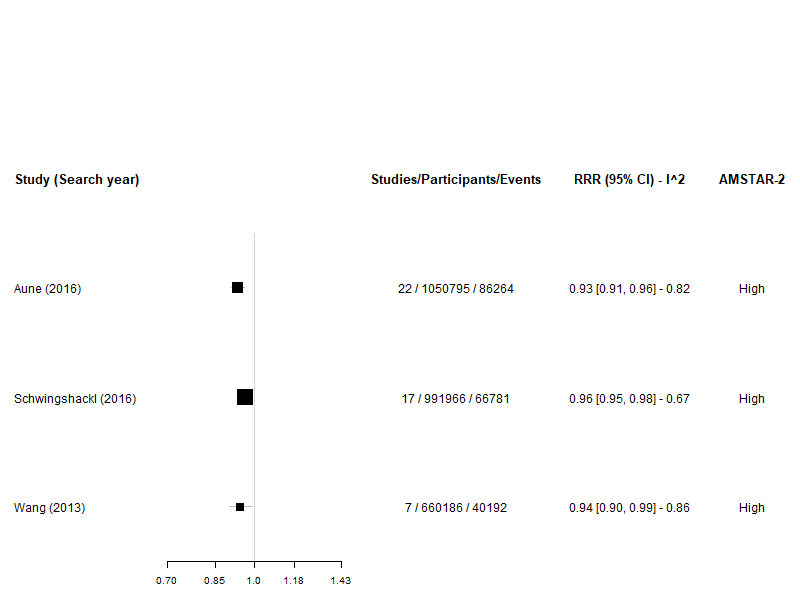


Supplementary Figure 9: Associations between intake of nuts for high versus low and all-cause mortality from all meta-analyses HL_Nuts_ACM


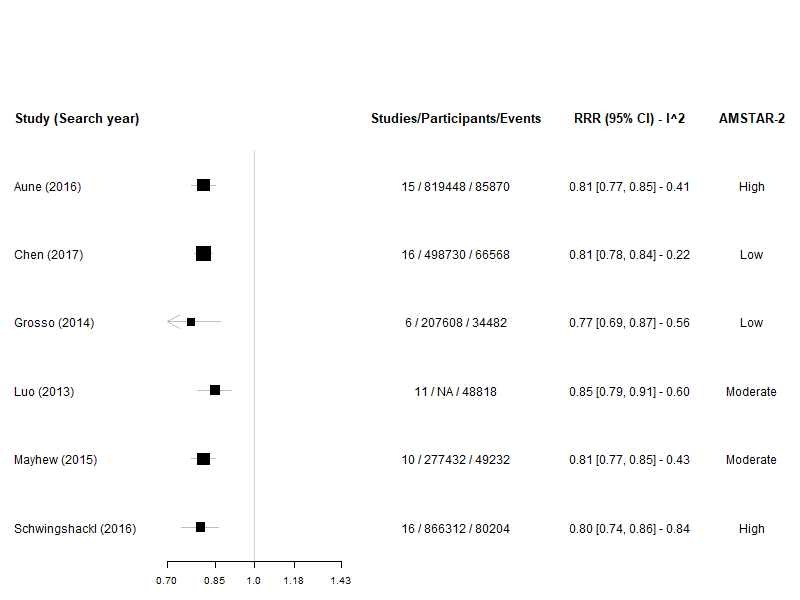


Supplementary Figure 10: Associations between intake of nuts per serving and all-cause mortality from all meta-analyses PS_Nuts_ACM

**
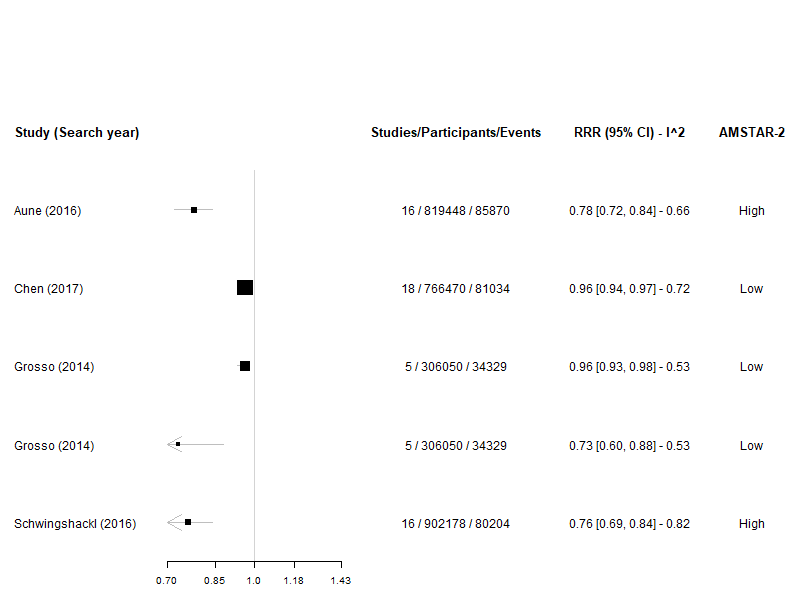
**

Supplementary Figure 11: Associations between intake of legumes for high versus low and all-cause mortality from all meta-analyses HL_Legumes_ACM

**
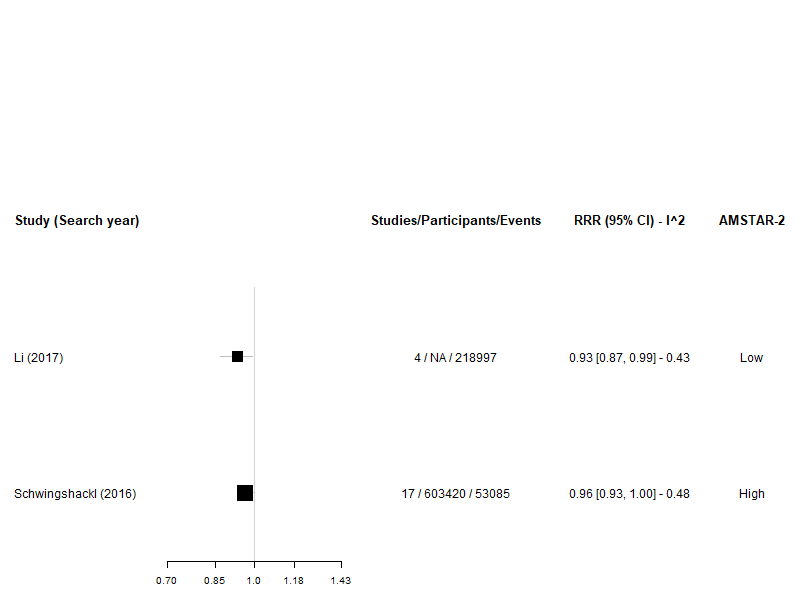
**

Supplementary Figure 12: Associations between intake of legumes per serving and all-cause mortality from all meta-analyses PS_Legumes_ACM

**
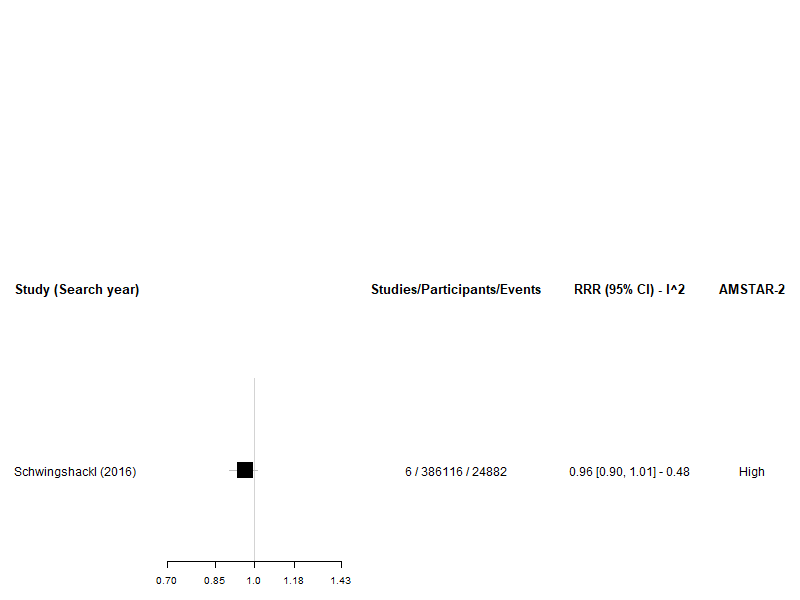
**

Supplementary Figure 13: Associations between intake of fish and fish products for high versus low and all-cause mortality from all meta-analyses HL_Fish_ACM

**
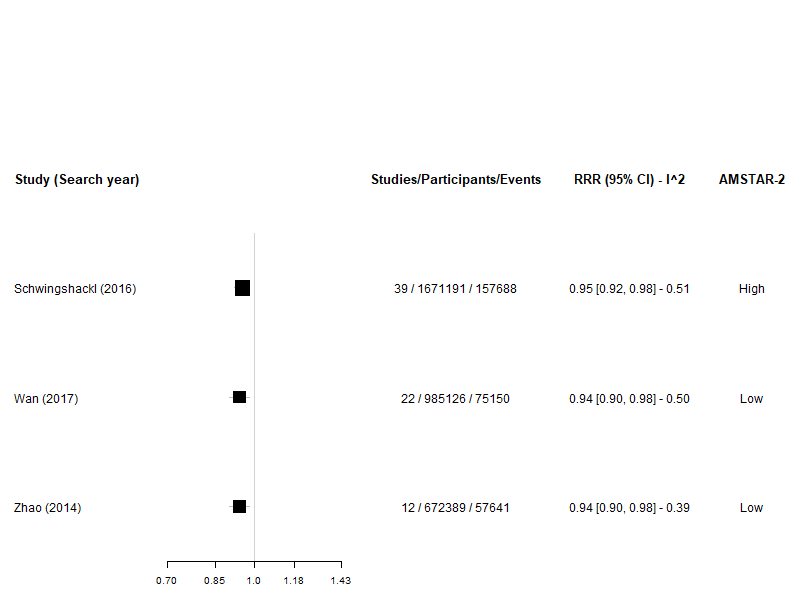
**

Supplementary Figure 14: Associations between intake of fish and fish products per serving and all-cause mortality from all meta-analyses PS_Fish_ACM

**
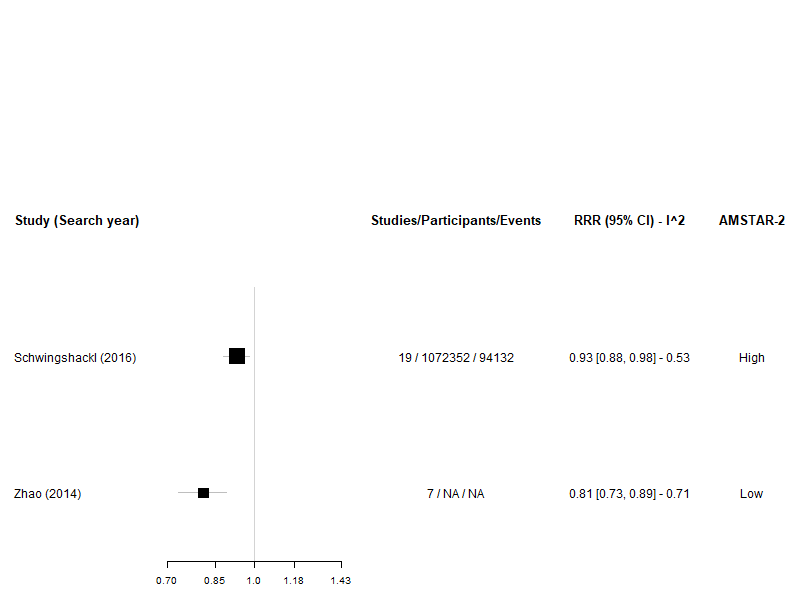
**

Supplementary Figure 15: Associations between intake of eggs for high versus low and all-cause mortality from all meta-analyses HL_Eggs_ACM

**
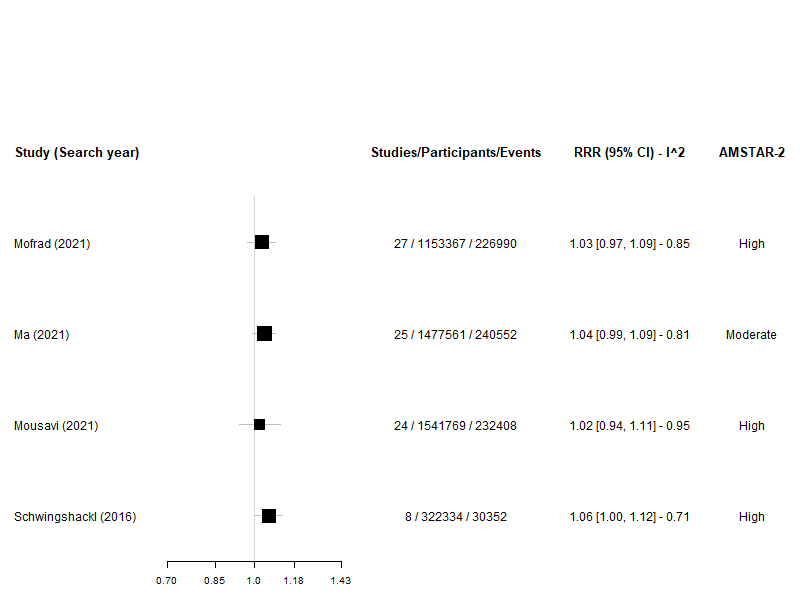
**

Supplementary Figure 16: Associations between intake of eggs per serving and all-cause mortality from all meta-analyses PS_Eggs_ACM


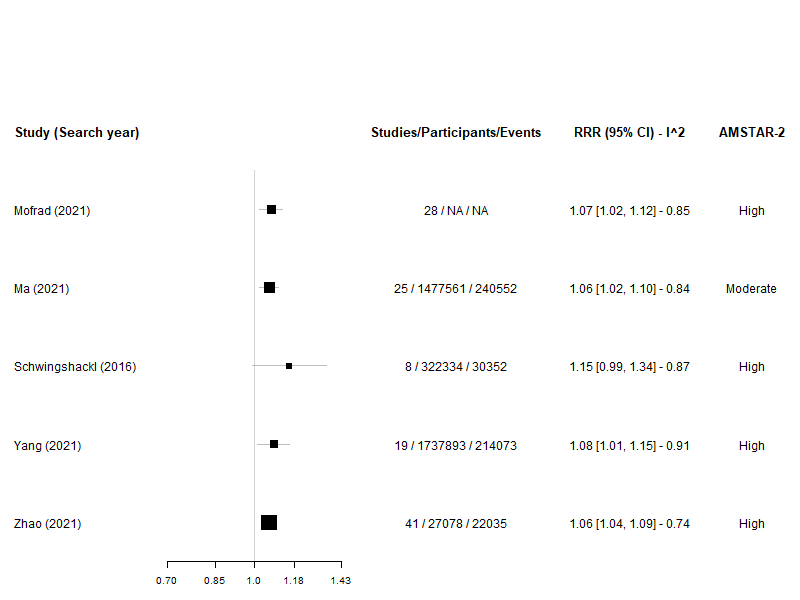


Supplementary Figure 17: Associations between intake of dairy products for high versus low and all-cause mortality from all meta-analyses HL_Dairy_ACM


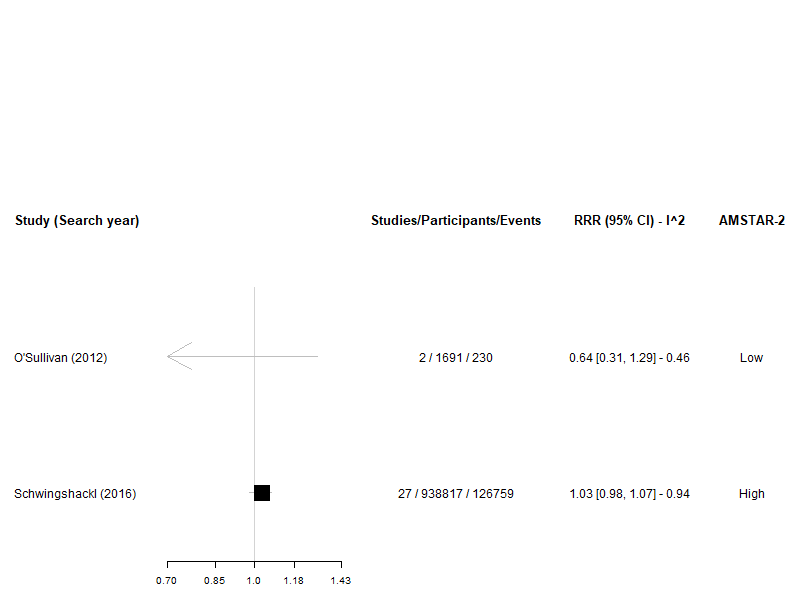


Supplementary Figure 18: Associations between intake of dairy products per serving and all-cause mortality from all meta-analyses PS_Dairy_ACM


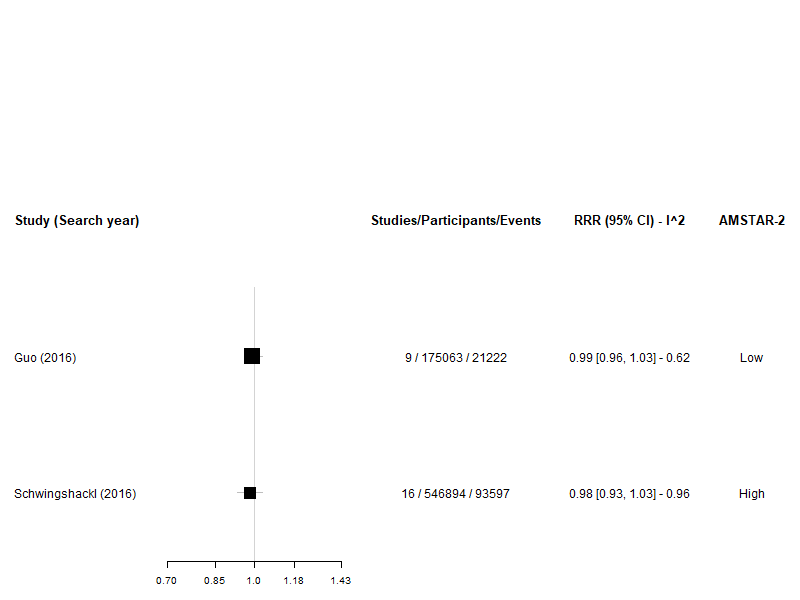


Supplementary Figure 19: Associations between intake of processed meat for high versus low and all-cause mortality from all meta-analyses HL_Processed meat_ACM


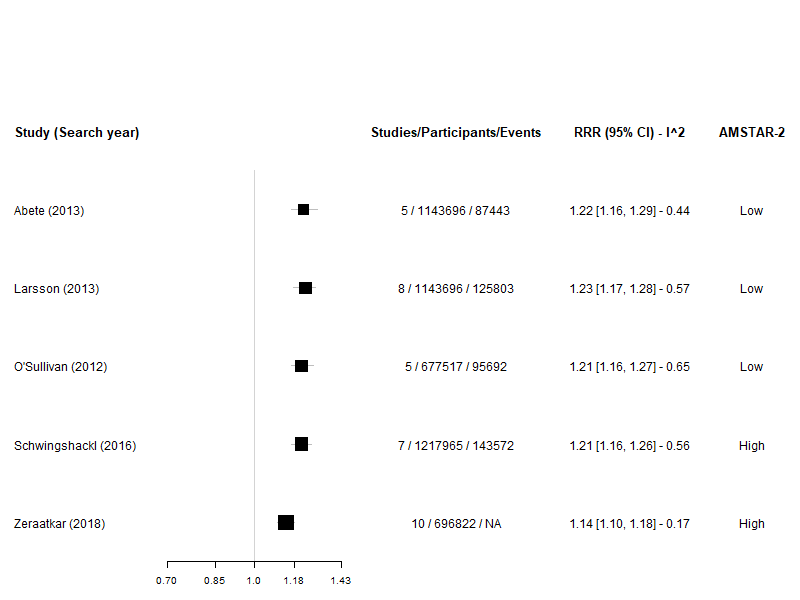


Supplementary Figure 20: Associations between intake of processed meat per serving and all-cause mortality from all meta-analyses PS_Processed meat_ACM


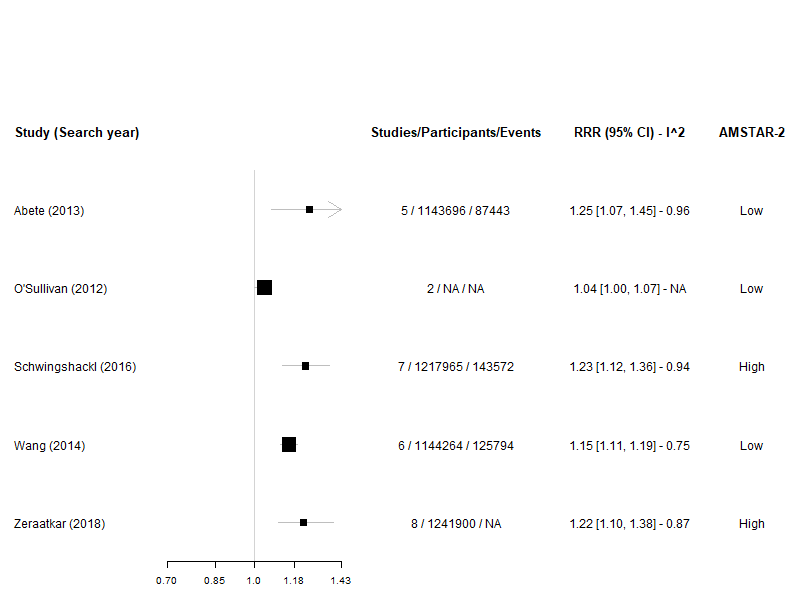


Supplementary Figure 21: Associations between intake of unprocessed red meat for high versus low and all-cause mortality from all meta-analyses HL_Unprocessed Red meat_ACM
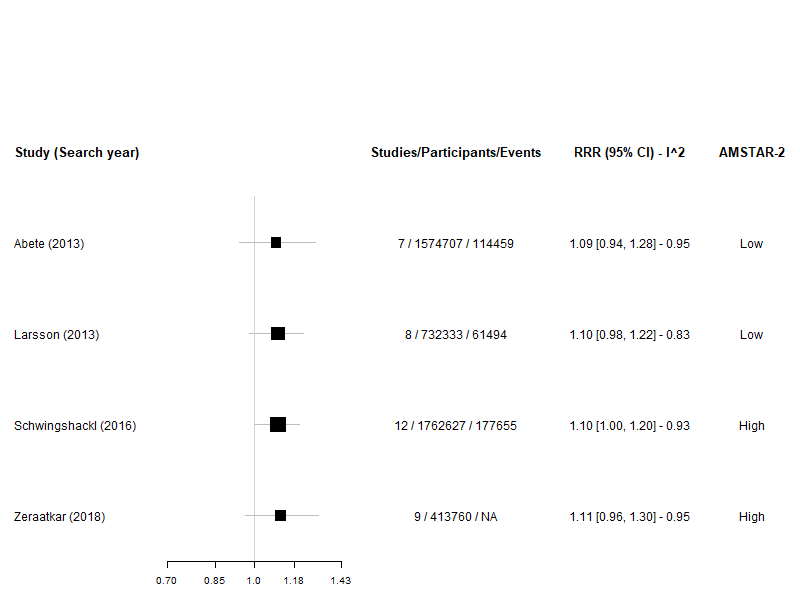


Supplementary Figure 22: Associations between intake of unprocessed red meat per serving and all-cause mortality from all meta-analyses PS_Unprocessed_Red meat_ACM
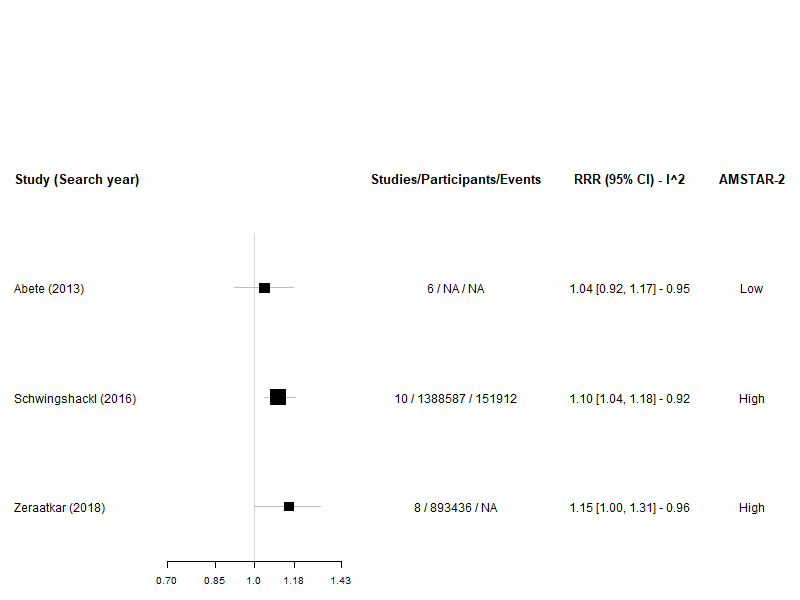


Supplementary Figure 23: Associations between intake of unprocessed white meat for high versus low and all-cause mortality from all meta-analyses HL_Unprocessed_White meat_ACM
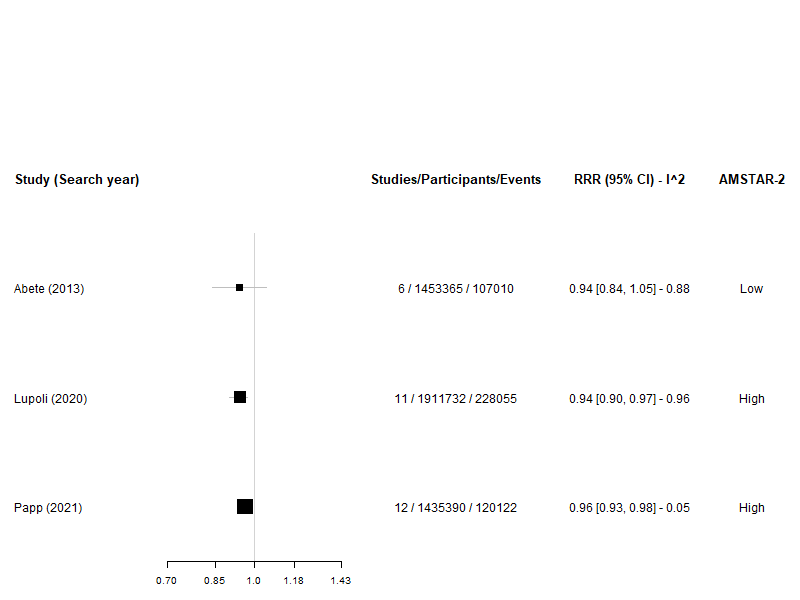


Supplementary Figure 24: Associations between intake of unprocessed white meat per serving and all-cause mortality from all meta-analyses PS_Unprocessed_White meat_ACM
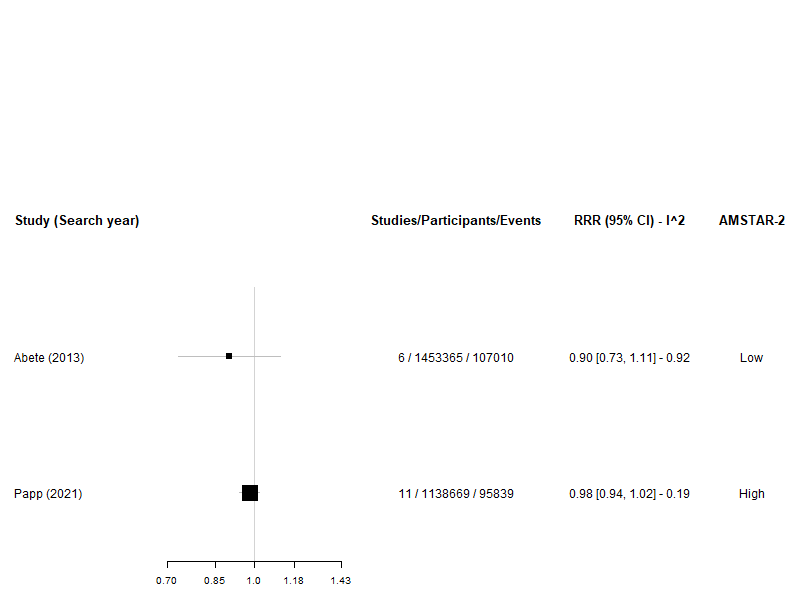


Supplementary Figure 25: Associations between intake of sugar-sweetened beverages for high versus low and all-cause mortality from all meta-analyses HL_SSB_ACM
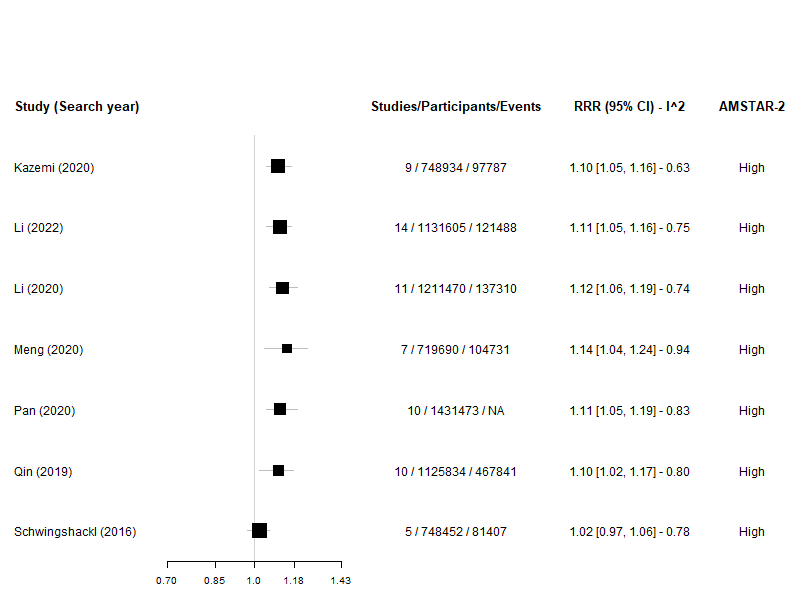


Supplementary Figure 26: Associations between intake of sugar-sweetened beverages per serving and all-cause mortality from all meta-analyses PS_SSB_ACM
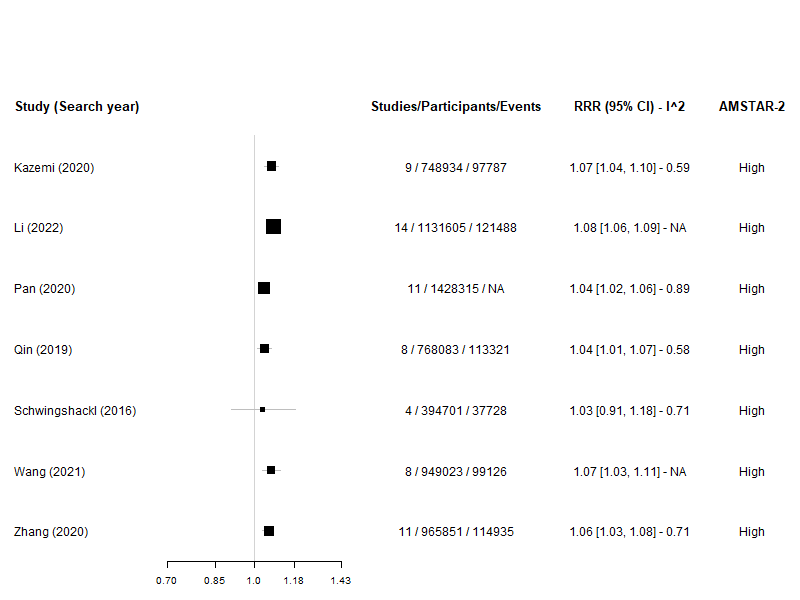


Supplementary Figure 27: Associations between intake of added sugars for high versus low and all-cause mortality from all meta-analyses HL_Added sugars_ACM
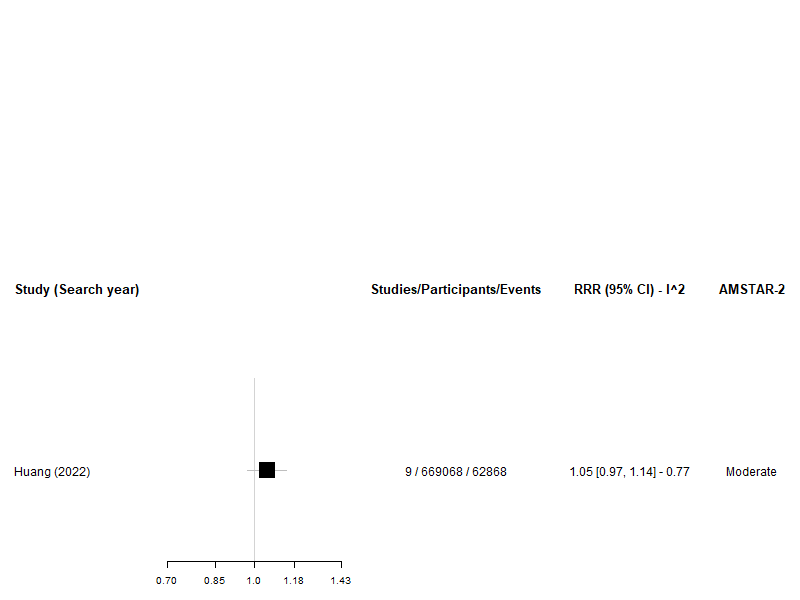


Supplementary Figure 28: Associations between intake of added sugars per serving and all-cause mortality from all meta-analyses PS_Added sugars_ACM
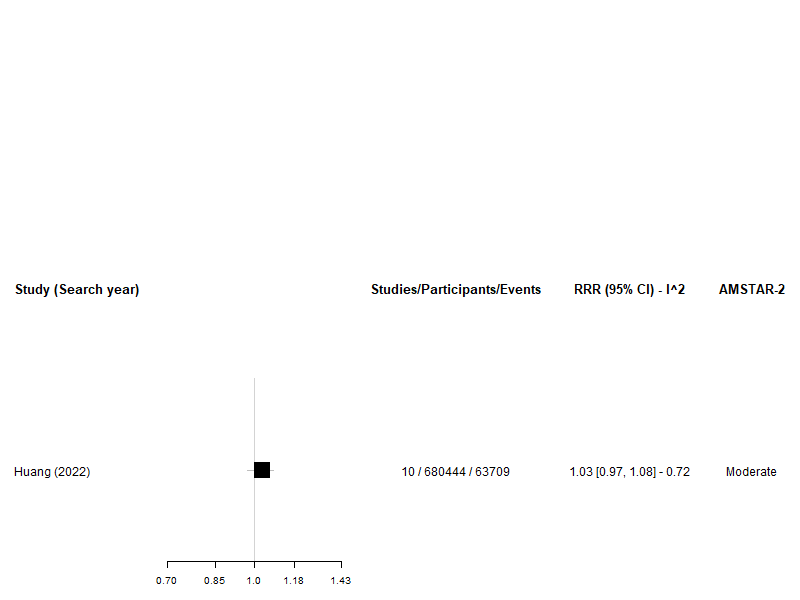

Supplement: multimedia component 2 [file mmc2.docx]
